# Spawning Dynamics and Size Related Trends in Reproductive Parameters of Southern Bluefin Tuna, *Thunnus maccoyii*

**DOI:** 10.1371/journal.pone.0125744

**Published:** 2015-05-18

**Authors:** Jessica. H. Farley, Tim L. O. Davis, Mark V. Bravington, Retno Andamari, Campbell R. Davies

**Affiliations:** 1 Oceans and Atmosphere Flagship, Hobart, Tasmania, Australia; 2 Digital Productivity Flagship, Hobart, Tasmania, Australia; 3 Institute for Mariculture Research and Development, Gondol, Bali, Indonesia; North Carolina State University, UNITED STATES

## Abstract

Knowledge of spawning behaviour and fecundity of fish is important for estimating the reproductive potential of a stock and for constructing appropriate statistical models for assessing sustainable catch levels. Estimates of length-based reproductive parameters are particularly important for determining potential annual fecundity as a function of fish size, but they are often difficult to estimate reliably. Here we provide new information on the reproductive dynamics of southern bluefin tuna (SBT) *Thunnus maccoyii* through the analysis of fish size and ovary histology collected on the spawning ground in 1993–1995 and 1999–2002. These are used to refine previous parameter estimates of spawning dynamics and investigate size related trends in these parameters. Our results suggest that the small SBT tend to arrive on the spawning ground slightly later and depart earlier in the spawning season relative to large fish. All females were mature and the majority were classed as spawning capable (actively spawning or non-spawning) with a very small proportion classed as regressing. The fraction of females spawning per day decreased with fish size, but once females start a spawning episode, they spawned daily irrespective of size. Mean batch fecundity was estimated directly at 6.5 million oocytes. Analysis of ovary histology and ovary weight data indicated that relative batch fecundity, and the duration of spawning and non-spawning episodes, increased with fish size. These reproductive parameter estimates could be used with estimates of residency time on the spawning ground as a function of fish size (if known) and demographic data for the spawning population to provide a time series of relative annual fecundity for SBT.

## Introduction

Total egg production is considered a more accurate representation of the reproductive potential of a species than spawning stock biomass as the size (or age) composition of the spawning population, and changes in annual fecundity with fish size/age, are taken into account in the former [1, 2]. An understanding of the relationships between reproductive parameters, such as spawning frequency, batch fecundity and spawning duration, with fish length are required to estimate potential annual fecundity at size for a multiple-spawning species with indeterminate fecundity. These data are rarely available for highly migratory species, such as tunas, and are particularly difficult to estimate reliably for species where individuals migrate to and from spawning areas at different times during the spawning season [3, 4].

Southern bluefin tuna (SBT) *Thunnus maccoyii* is a large, long-lived pelagic species which feed in the temperate waters of the Southern Ocean [5–7]. After reaching maturity at age ~10–12 years, adults undertake annual migrations between these feeding grounds and a single spawning ground in the northeastern Indian Ocean [8, 9]. The spawning stock of SBT has been monitored since the early 1990s through a catch-monitoring program of the Indonesian longline fishery on the spawning ground [10]. The program detected changes in the size and age composition of fish caught in the early 2000s; the abundance of small/young fish increased relative to large/old fish [11]. Monitoring the SBT spawning stock was imperative as the species is considered to be overfished and the spawning stock is at historically low levels [12, 13] following substantial fishing pressure since the 1950s.

The Indonesian catch-monitoring program provided biological samples from SBT between 1992 and 1995 to study their reproductive dynamics [8]. The study collected ovaries from females predominantly between 165 and 195 cm fork length (*FL*) and found that all were mature adults that were capable of spawning, except for a few regressing fish that had just completed spawning (<1% of females examined). The population spawning season was protracted (effectively from September to April) although individuals did not spawn for that entire period and departed as soon as spawning was completed, to be replaced by newly arriving fish [8]. The majority of fish were classified as either actively spawning (ovary contained evidence of spawning activity such as oocytes at the migratory nucleus or hydrated stage, or postovulatory follicles) or non-spawning (ovary contained tertiary vitellogenic oocytes but no evidence of spawning activity). Those that were spawning released 6 million eggs on average per day. It was proposed that fish in non-spawning mode were either recovering from the energetic costs of migration before spawning, or were resting between spawning episodes [8]. Although regressing (post-spawning) fish were rare [8], they were distinguished from non-spawning fish by high levels of atresia or both early and advanced yolked oocytes, or no yolked oocytes present in the ovary. Farley and Davis [8] provided the first quantitative understanding of the dynamics of spawning of SBT; however, questions remained regarding the duration of spawning and non-spawning episodes, and annual residency time on the spawning ground. In addition, very little information was obtained on the smaller SBT that appear on the spawning ground. It is these fish that are in the process of recruiting into the spawning population and are likely to provide influential data on the relationship between fish size and egg production.

A second field program was completed between 1999–2002 that provided the samples needed to refine previous estimates of spawning parameters and investigate size related trends in some of these parameters. The increased number of small SBT in the catches on the spawning ground from the early-2000s meant that it was also possible to obtain sufficient numbers of small fish to improve parameter estimation for these smaller size classes.

## Methods

### Ethical statement

Ethical approval was not required for this study, as all fish were collected as part of routine fishing procedures. No samples were collected by the authors. All samples in this study originated from the Indonesian longline fishery and were already dead when sampled as part of commercial processing operations. Fish were sacrificed by the commercial fisher at sea using standard fisheries practices. Permission was granted to use samples from all fish. All samples were donated. No field permits were required to collect samples, since all originated from commercial catch. SBT are not a protected species in any ocean.

### Biological sampling and processing

Biological samples were obtained from 640 SBT caught on their spawning ground by the Indonesian longline fishery operating out of Benoa, Bali between 1999 and 2002. Ovaries were sampled from fresh fish (held on ice) that were landed at export processing facilities in Benoa. *FL* (cm) and eviscerated body weight (*BW*, g) were measured for all fish. Ovaries were trimmed of extraneous fat and tissue and weighed to the nearest gram. Gonad index (*GI*) was as *GI = GW/FL* ^*3*^ *x 10* ^*4*^ where *GW* is ovary weight in g [8]. A 12 mm diameter core sub-sample was taken from each ovary and fixed in 10% buffered formalin for subsequent histological processing. Ovaries from fish that were close to spawning and were possible candidates for estimating batch fecundity (i.e. contained oocytes at the hydrated stage) were frozen.

To examine changes in body condition, the relative condition factor (*Kn*) [14] was calculated using *Kn = BW/ aFL* ^*b*^ where *a* and *b* are the intercept and slope parameters estimated from the natural log (*ln*) transformed *FL-BW* linear relationship estimated from the data. Analysis of variance (ANOVA) was used to test for statistically significant differences in *FL*, *GI* and *Kn* among spawning conditions and months.

### Histological classification

Standard histological sections were prepared from the fixed ovarian tissue (cut to 6 μm and stained with Harris’ haematoxylin and eosin). Ovaries were classified using criteria similar to that developed for other pelagic species including tunas [15–18], standardised terminology for classifying fish reproduction [19]. The most advanced group of oocytes (MAGO) present in each ovary was staged into one of 5 classes: unyolked (primary growth and cortical alveolar), early yolked (primary and secondary vitellogenic), advanced yolked (tertiary vitellogenic), migratory nucleus (germinal vesicle migration) or hydrated. Each ovary was also scored according to the presence and age of postovulatory follicles (POFs). Postovulatory follicles were aged according to their state of degeneration using criteria developed for skipjack *Katsuwonus pelamis*, yellowfin *T*. *albacares* and bigeye tuna *T*. *obesus* [17–21] all of which spawn in water temperatures above 24°C and resorb their POFs within 24 hours of spawning. It was assumed that SBT resorb POFs at the same rate as other tropical spawning tuna as water temperature appears to be the dominant factor governing resorption rates [22]. Postovulatory follicles were staged as: absent, new, <12 hours old, 13–24 hours old or indistinguishable (due to tissue decay). Finally, each ovary was classified by the level of α and β stage atresia of advanced yolked oocytes present (atresia is the process of resorbing oocyte). Four levels of α stage atresia were recorded: <10% (minor atresia), 10–50% (moderate), >50% (major), 100% (complete). The β stage of atresia involves the remaining granulosa and thecal cells being reorganised and resorbed leaving a compact structure containing several intercellular vacuoles. This stage was recorded as being present or absent.

All females on the spawning ground were mature and were classified into reproductive phases and subphases depending on the MAGO, POF and atresia present in the ovary.

**Fig 1:**
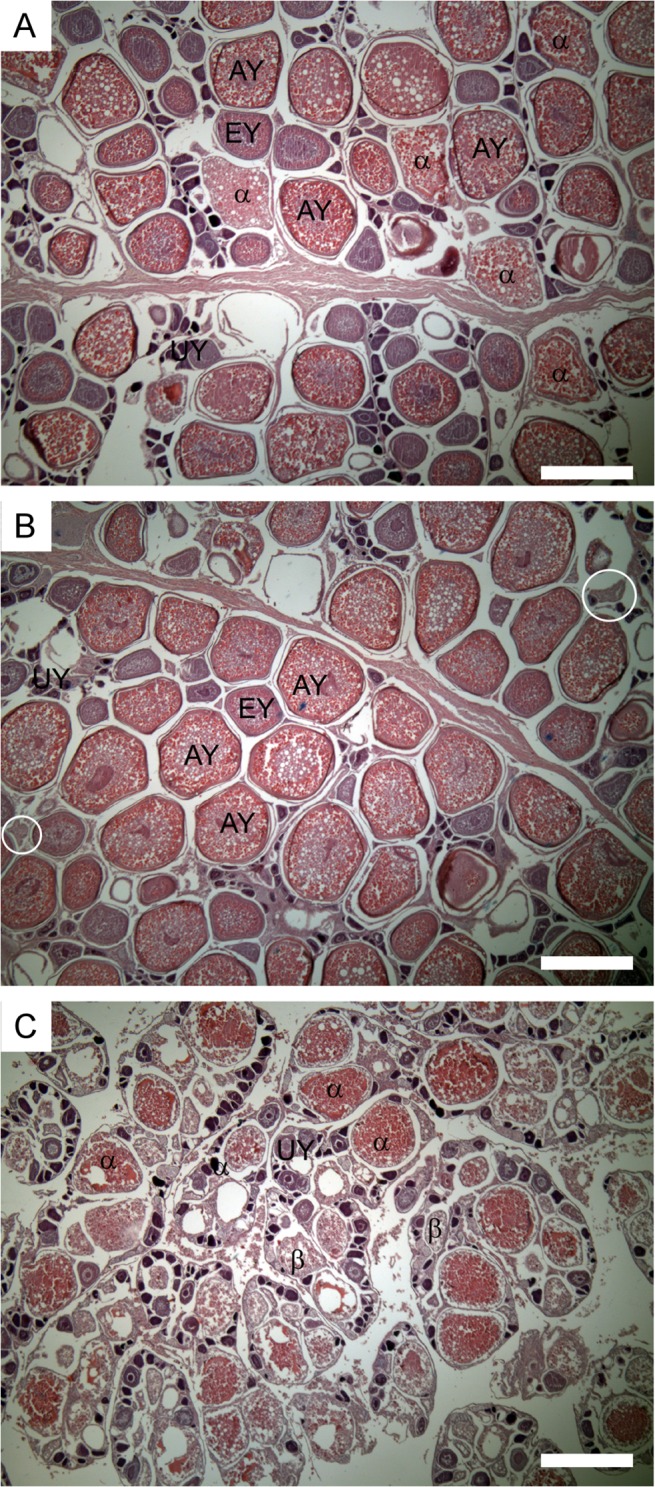
Histological sections of ovaries showing examples of the reproductive phases. (A) non-spawning female with advanced yolked (AY) oocytes, alpha (α) atresia, but no evidence of imminent or recent spawning activity; (B) actively spawning female with AY oocytes, post-ovulatory follicles (POFs) but no α atresia; (C) regressing female with unyolked (UY) oocytes and massive atresia of AY oocytes. EY = early yolked oocytes, β = beta atresia. White circles indicate postovulatory follicles. The white scale bars is 500 μm.

1. Spawning capable phase:
  1.1 Non-spawning subphase—ovary contains advanced yolked oocytes but no evidence of imminent or recent spawning activity (migratory nucleus or hydrated oocytes or POFs). Atresia of advanced yolked oocytes may be present. Fig 1A.
  1.2 Actively spawning subphase—ovary contains advanced yolked oocytes and evidence of imminent (migratory nucleus or hydrated oocytes) or recent (POF) spawning activity. Atresia of advanced yolked oocytes may be present. Fig 1B.
2. Regressing phase: ovary contains unyolked oocytes and all advanced yolked oocytes are in the α or β stages of atresia. Fig 1C.

### Spawning frequency

Spawning frequency was estimated by the postovulatory follicle method [23]. This method uses the incidence of mature females with POFs (assumed to remain visible for only 24 hours in SBT ovaries) to estimate the fraction of the population spawning per day (spawning fraction) and spawning frequency (inverse of spawning fraction). The spawning fraction and spawning frequency were determined from spawning capable fish only; regressing fish were rarely encountered (only five during both studies) and were not included in the calculation.

### Batch fecundity

Histology was used to determine whether ovaries were suitable for determining batch fecundity—ovaries were selected that contained hydrated oocytes but did not have new POFs, which would indicate partial spawning of the batch. The thawed ovary was reweighed to the nearest gram, and two sub-samples were removed from each lobe of the ovary. The sub-samples, each about 0.5g – 1.0g in weight, were cores through the entire ovary from the periphery to the lumen. These were weighed to the nearest 0.01 mg, fixed in 10% buffered formalin, and the number of hydrated oocytes counted following [8]. The number of hydrated oocytes per gram of ovary sub-sample was raised to the weight of both ovaries to give an estimate of batch fecundity.

An indirect method to estimate changes in batch fecundity with size was developed based on the difference in *GW* before and after spawning. “Before” ovaries were classified as ovaries with hydrated oocytes and no new POFs (to exclude partial spawning events), and “after” ovaries were classified as ovaries with advanced yolk oocytes and new or <12 hour old POFs. A Gamma GLM with log-link was fitted to data for all “before” and “after” fish, where the intercept depends on before/after status but slope does not, i.e.:

$$\begin{array}{l} \text{log E}\;\left[{\text{w}}_{l\text{B}}\right]=\beta +\gamma \;\text{log}\;l\\ \text{log E}\;\left[{\text{w}}_{l\text{A}}\right]=\alpha +\gamma \;\text{log}\;l \end{array}$$

for OW, *w*_*l*_, at fork length l, before/after indicated by *B*/*A*, and parameters *α*, *β* and *γ* to be estimated. Given this formulation, the relative batch fecundity of length $l_{2}$ compared to length $l_{1}$ is given by:

$$\frac{w_{l_{1}B}-w_{l_{1}A}}{w_{l_{2}B}-w_{l_{2}A}}={\left(\frac{l_{1}}{l_{2}}\right)}^{\gamma }$$

### Duration of spawning and non-spawning episodes

The number of days of sequential spawning by an individual can be estimated from samples of spawning fish if the fraction spawning on their first day can be identified. The reciprocal of that fraction is the average number of days of sequential spawning. Fish with migratory nucleus oocytes indicate imminent hydration and spawning. When such fish lack POFs, then they could not have spawned within the previous 24 hours and, hence, they are in the first day of their spawning cycle. A binomial GLM was fitted to estimate the proportion of migratory oocyte stage ovaries with no POFs as a function of fish length. Fish with hydrated oocytes were not included in the analysis because by that stage of the daily cycle, it is unlikely that POFs would still remain in the ovaries. Missing the presence of POFs would result in a negative bias of the number of daily spawning events.

Of the fish with advanced yolked oocytes, those with POFs 13–24 hours old must have spawned recently but do not appear to be maturing a new batch of oocytes. That is, they are likely to be in the first day of their resting cycle. Again, a binomial GLM was used to estimate the proportion of females with advanced yolked stage ovaries that had just finished spawning as a function of fish length. The reciprocal of this proportion is the average number of days of sequential resting. Fish with advanced yolked oocytes and new or <12 hour POF were not included in the analysis because it was unknown whether these fish would develop a new batch of oocytes within the following 12–24 hours. It is unlikely that females with 13–24 hour POFs would not be undertaking final oocyte maturation (i.e. the development of migratory nucleus of hydrated oocytes) indicating imminent spawning. Migratory nucleus oocytes are observed as early as 10:30am to 13:00pm in tunas such as yellowfin tuna and albacore tuna (*T*. *albacores*) [24–26].

## Results

### Spawning behaviour

Data obtained for the 640 females sampled in the current study were combined with data from 475 individuals from [8] providing a total of 1,115 ovaries (S1 Table). The majority of fish sampled were 150–210 cm *FL* although length information was only available for 65.4% of females sampled in the first study. The estimates of the *ln*(a), *a* and *b* parameters of the *FL-BW* relationship were -7.28 (SE = 0.27), 6.89x10^-4^ and 2.30 (SE = 0.05) respectively.

**Fig 2:**
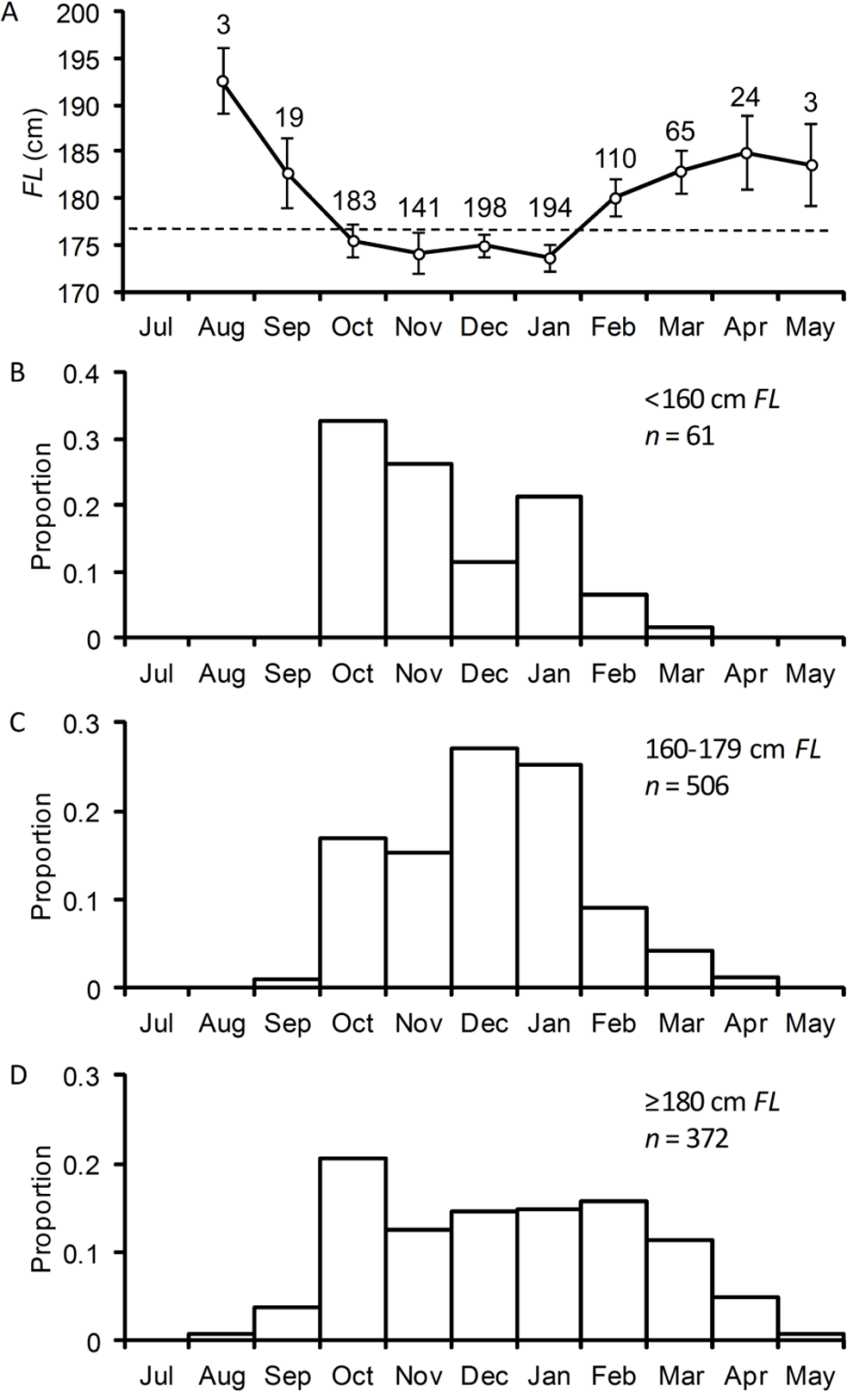
Seasonal variation in the size of southern bluefin tuna caught on the spawning ground. (A) Mean *FL* ± 2 S.E. by month; sample sizes are shown above the mean and the dashed line indicates the mean for all females (176.3 cm *FL*). (B-D) Proportion of fish sampled by month for three length class.

The mean size of females sampled varied with month (ANOVA: *P* < 0.001). Females sampled in the first and last months of the spawning season (August/September and April/May) were larger on average than those sampled in the middle months of October to January (Fig 2A). When examined by length class, the smallest females (<160 cm *FL*) were predominantly sampled over a four-month period between October and January, (Fig 2B). Females 160–179 cm *FL* were sampled over the same four month period, but a proportion were caught in February to April (Fig 2C). The largest females (≥180 cm *FL*) were sampled over a broader 6 month period from October through to March, with a small proportion also caught both prior to (August and September) and after (April and May) the main spawning period (Fig 2D).These results suggest that the smaller individuals tend to arrive slightly later and depart earlier in the spawning season than the largest individuals.

**Fig 3:**
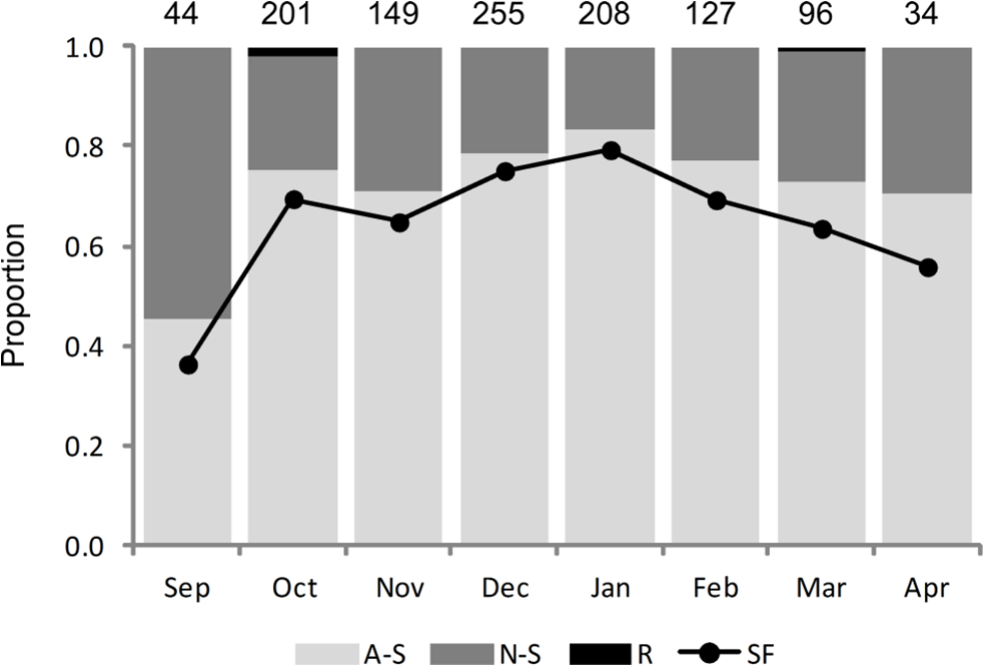
Proportion of females in each reproductive phase and spawning fraction by month. A-S = actively spawning, N-S = non-spawning, R = regressing, SF = spawning fraction. Sample sizes are indicated at the top. The results are only shown if n ≥ 5.

All females were mature; 1110 were spawning capable and 5 were regressing (Table 1). Of the spawning capable females, 75.3% were actively spawning, 23.9% were non-spawning and 0.8% were unknown due to tissue decay. The majority (87.9%) of actively spawning females had minor levels of atresia in their ovaries, while the majority (82.6%) of non-spawning females had moderate levels of atresia (Table 1). Spawning capable females with major (>50%) atresia were rare with only 2–3 sampled per month in October to February; these fish were likely to be near the end of their spawning seasons. The relative abundance of actively spawning females was lowest at the beginning of the spawning season (September) and highest in the middle of the season (December to February) (Fig 3). In contrast, the relative abundance of non-spawning females was highest in September and lowest in January, but comprised a relatively stable fraction (16.8 to 29.4%) in October to April (Fig 3). Although scarce, regressing females with 100% atresia (n = 5) were sampled in October and March (Fig 3). The presence of low number of these early post-spawning and regressing females throughout the spawning season confirms that spawning is not synchronised for SBT and that they leave the spawning ground immediately after completing their spawning cycle for the season (Fig 3).

**Table 1:** Number of females classified by reproductive phase/subphase and level of alpha stage atresia (α).

Phase	Subphase	Minor α (<10%)	Moderate α (10–50%)	Major α (>50%)	Complete α (100%)	Total
Spawning capable	Spawning	735	96	5		836
	Non-spawning	38	219	8		265
	Unknown^1^	2	7			9
Regressing					5	5
Total		775	322	13	5	1115

^1^ Reproductive phase could not be determined because the ovaries contained advanced yolked oocytes but postovulatory follicles were indistinguishable due to tissue decay.

**Fig 4:**
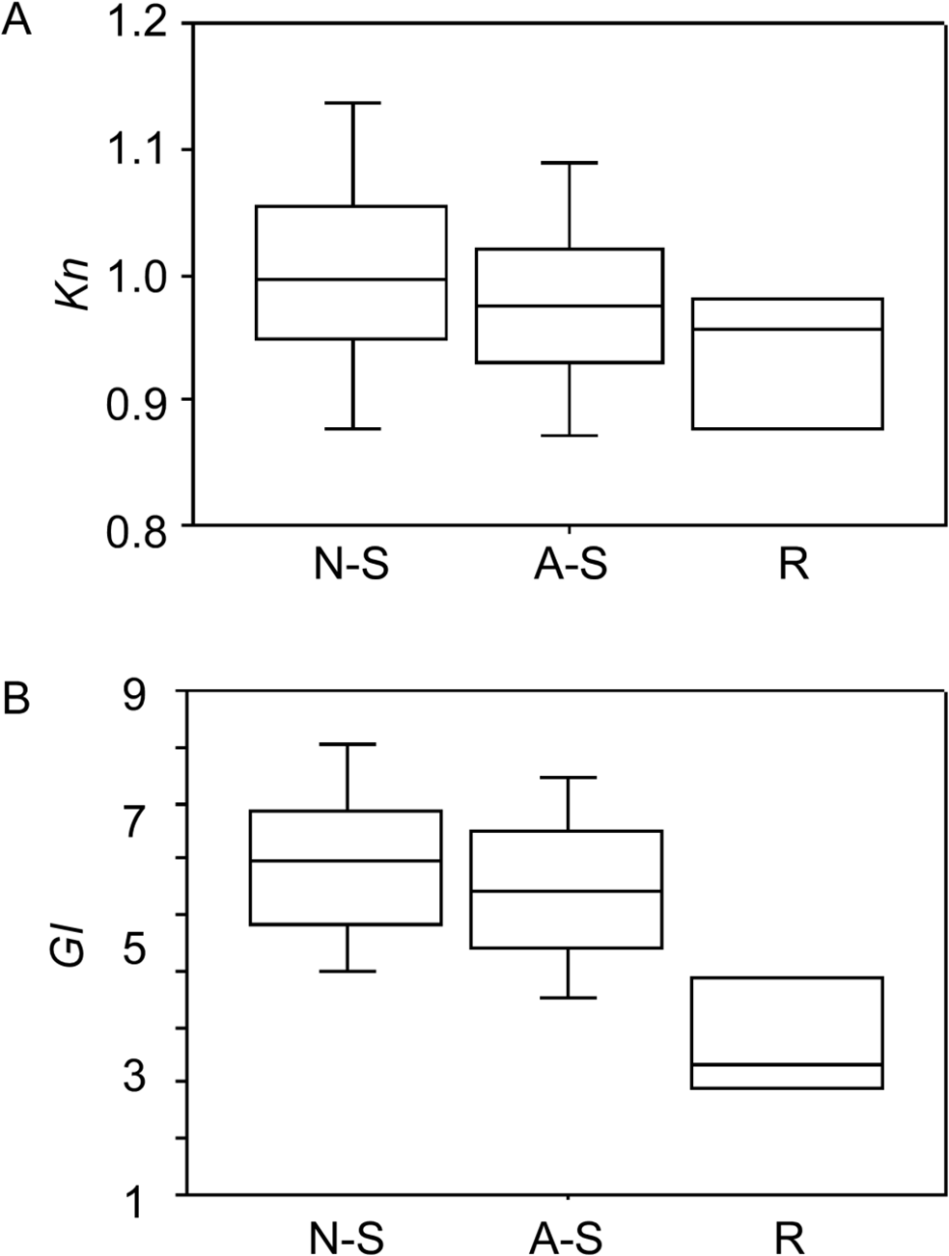
Variation in relative condition factor (*Kn)* (A) and gonad index (*GI*) (B) with reproductive phase. N-S = non-spawning, A-S = actively spawning, and R = regressing. Females with hydrated oocytes were excluded from the GI analysis. The box represents the median and interquartile range, and the vertical lines represent the 95% confidence interval for the median.

**Fig 5:**
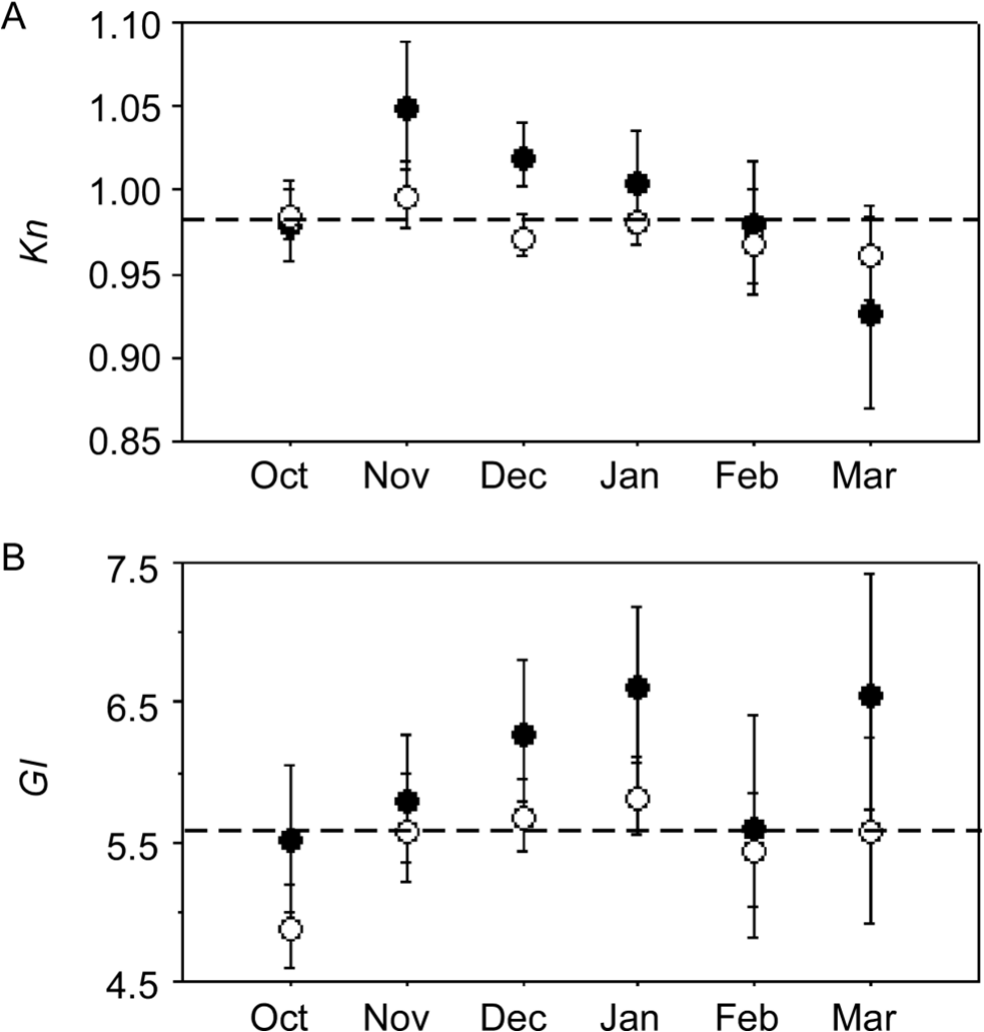
Seasonal variation in mean relative condition factor (*Kn*) (A) and gonad index (*GI*) (B). Non-spawning females = ●, actively spawning females = ○. Results are not shown for months where n ≤ 10. Females with hydrated oocytes were excluded from the GI analysis to remove the effect of heavy hydrating ovaries. Dashed line indicates the mean for all females. Error bars are ± 2 S.E.

The relative condition (*Kn*) and gonad index (*GI*) of females varied with reproductive phase (ANOVA: *P* < 0.01). Non-spawning females had the highest *Kn* and *GI* on average, followed by actively spawning and regressing females (Fig 4). When examined by month, mean *Kn* was higher for non-spawning than spawning fish in most months (Fig 5A) although ANOVA indicated significant differences in November and December only (ANOVA; *P* < 0.01). *Kn* was highest in November and generally declined towards the end of the spawning season (Fig 5A).Mean *GI* was highest for non-spawning than spawning females in all months (Fig 5B) although ANOVA indicated significant differences in October, December and January only (*P* < 0.05) (Fig 5B). Overall, mean *GI* was low in October and then increased until January, before declining in February.

### Spawning frequency

The estimated mean spawning fraction was 0.70 giving a mean spawning interval of 1.4 days. The spawning fraction of females classed as actively spawning was 0.92 resulting in a mean spawning interval of 1.1 days. These data provide strong evidence that once females start spawning they spawn daily. The fraction of females spawning per day increased between September (0.37) and January (0.79) and then declined through to April (0.56) (Fig 3). The fraction of females spawning per day also varied with fish length. Females in the 150–169 cm, 170–189 cm and ≥190 cm length classes spawned on average every 1.3, 1.4 and 1.6 days respectively. When the data were restricted to spawning females, however, the mean spawning fraction was 1.1 days for all *FL* classes.

### Batch fecundity

**Fig 6:**
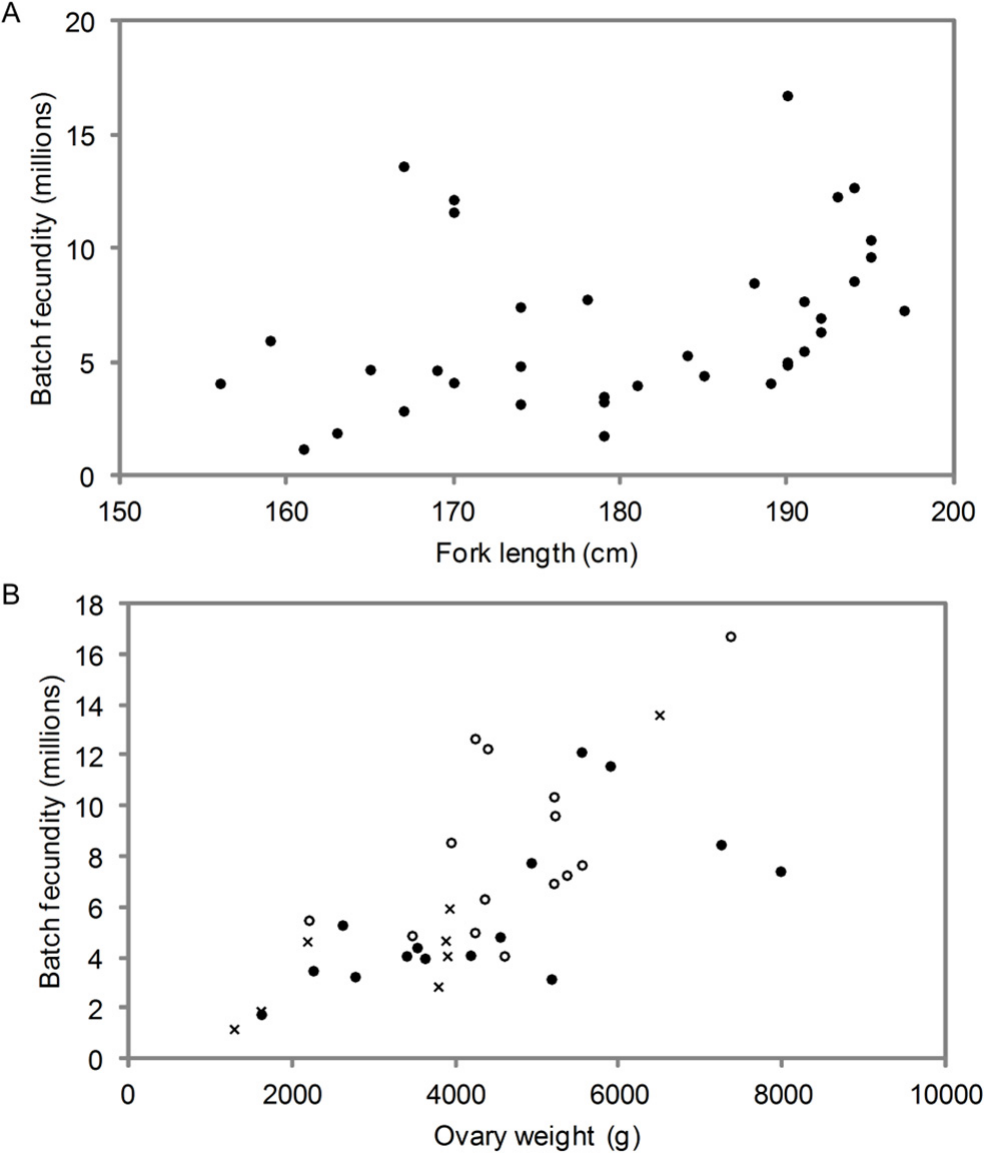
Relationship between batch fecundity and fork length (A) and ovary weight (B) (n = 36). Data for (B) are separated into three fork length classes; x = <170 cm, ● = 170–189 cm, ○ = ≥190 cm FL.

Batch fecundity was determined directly on 20 ovaries from the first study [8] and a further 16 ovaries in this study. Numbers were limited because of the strict criteria used to select ovaries suitable for determining batch fecundity—i.e. those containing hydrated oocytes and no evidence of egg loss (lack of new POFs). Mean batch fecundity for the combined data was 6.5 million oocytes. While there was an increase in estimated fecundity with length, the relationship was highly variable (Fig 6A). The wide scatter made it impossible to estimate the relationship with fish length with any precision; the point estimate of the slope of log (fecundity) on log (length) was 3.90 but with a standard error of 1.37. There was insufficient data to examine seasonal and inter-annual differences in fecundity of SBT.

**Fig 7:**
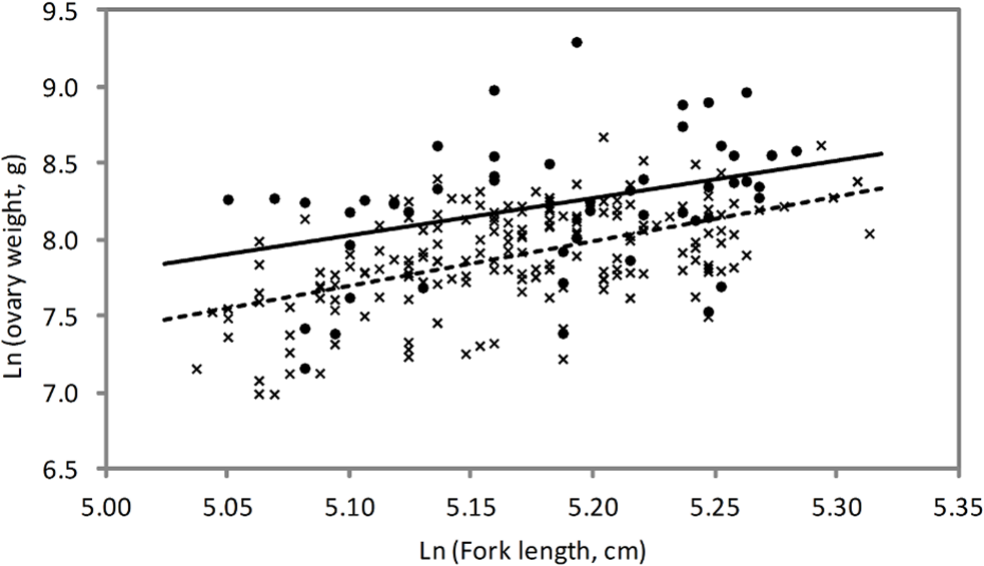
Relationship between ovary weight and fork length (natural log transformed) for southern bluefin tuna. Crosses/dotted line are ovaries with advanced yolked oocytes and new or <12 hour POFs (just spawned—*after* ovaries; n = 176), and dots/solid line are ovaries with hydrated oocytes without new POFs (about to spawn—*before* ovaries; n = 52).

The small sample size and high variability in directly estimated batch fecundity made it necessary to use the alternative method to estimate the relationship between fecundity and fish size. This was done by taking the difference between *GW* before and after spawning and examining the relationship with length. There was a strong relationship between batch fecundity and *GW* (Fig 6B). This could be due to larger ovaries containing more eggs and consequently larger batches of eggs are produced each spawning, and that hydrating eggs contribute a significant amount to the weight of the ovary. A comparison of the weight of ovaries of fish that were about to spawn (“before” ovaries) with those that had recently spawned (“after” ovaries) showed that for fish of similar lengths, most “before” ovaries were much heavier than “after ovaries” (Fig 7), although there was some overlap in *GW* between the stages. Hydrated oocytes, therefore, do have a marked affect on the weight of the ovary during the daily spawning cycle. The point estimates of slopes on *ln*-length are very similar (about 2.45 before spawning versus 2.89 afterwards) although the confidence interval on before-spawners is wide because there were only 51 samples available. On this evidence and a priori grounds, it is reasonable (but not proven) to assume that *GW* increases by a constant proportion just before spawning, independent of fish length. There is a suggestion that variances are slightly higher for “before” fish (estimated residual standard error of 0.41 versus 0.31), but allowing for this would make only a minor difference on the point estimates and it was ignored. The point estimate of *γ* is 2.64 (SE = 0.33); since body weight is roughly proportional to $l^{3}$ (actually 2.91 according to [27]) and 2.64<3, this means that ovaries become lighter relative to body weight as (mature) fish continue to grow. The point estimate of *β* − *α* is 0.35 (SE = 0.05), indicating that about 42% of the hydrated *GW* is lost during each spawning event. Table 2 shows the corresponding relative batch fecundity across lengths, compared to a 190cm fish. Estimates of relative batch fecundity were consistent with the relationship between direct measurements of batch fecundity and *FL*; however it could be estimated more precisely.

**Table 2:** Estimated batch fecundity estimate relative to a 190 cm southern bluefin tuna.

Length (cm)	Point estimate	95% LCL	95% UCL
160	0.66	0.61	0.70
170	0.76	0.73	0.80
180	0.88	0.86	0.89
190	1.00	1.00	1.00

### Duration of spawning and non-spawning episodes

**Fig 8:**
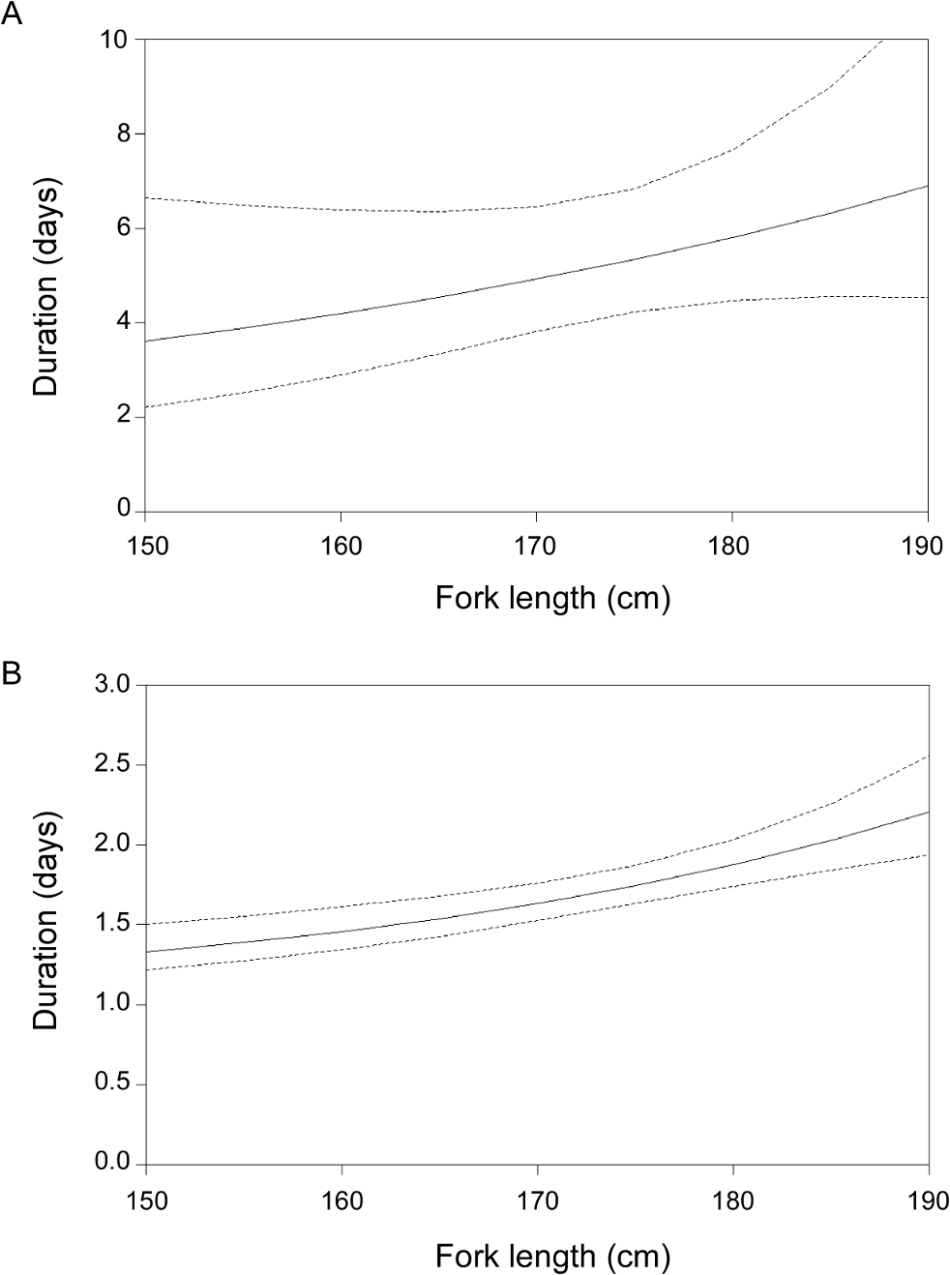
Relationship between fork length and spawning duration (A) and resting duration (B). 90% confidence intervals of the estimate are shown.

Based on migratory nucleus oocyte stage ovaries (*n* = 250) and the presence/absence of POFs (Table 3), the proportion of females sampled that did not spawn the day before capture (n = 40) was 0.16, providing a mean number of sequential daily spawning events for SBT at 6.25 days. There was a trend for increasing spawning duration with fish length, although the GLM was not statistically significant (*p* > 0.1) (Fig 8A). This model predicts 3.6 (SE = 1.4) sequential spawning events for a 150 cm fish and 6.9 (SE = 2.0) for a 190 cm fish.

**Table 3:** Number of ovaries classified by the most advanced group of oocytes and postovulatory follicle stage present.

MAGO	POFs absent	POFs new or <12 hr	POFs 13–24 hr	NA
AY	265	199	311	9
MN	40	12	198	1
H	24	12	37	1

MAGO = most advanced group of oocytes, AY = advanced yolked, MN = migratory nucleus, H = hydrated, NA = indistinguishable, POF = postovulatory follicle.

Based on ovaries with advanced yolked stage oocytes and either no or 13–24 hour POFs (n = 576) (Table 3), the proportion of females sampled that had just finished spawning (n = 311) was 0.54, providing a mean number of sequential resting days of 1.85. There is a trend for increasing resting duration with fish length, and the GLM was significant at (p <0.01) (Fig 8B). This model predicts 1.3 (SE = 0.1) sequential resting days for a 150 cm fish and 2.2 (SE = 0.2) sequential resting days for a 190 cm fish.

## Discussion

The combined data set obtained from the 1992–1995 and 1999–2002 programs provided important new information on the spawning behaviour of SBT and allowed exploration of size-related differences in reproductive parameters. This study found that large females are caught on the spawning ground over a longer period relative to smaller fish; large fish appeared as early as August/September albeit in relatively small numbers. The arrival of large fish before smaller fish in spawning areas has been documented in several pelagic and non-pelagic fish species, including Atlantic bluefin tuna (*T*. *thynnus*), and has been linked to size-related differences in migration, maturation and temperature preferences [28, 29]. Large SBT may have faster swimming speeds and can cover the migration routes from the Southern Ocean in a shorter time compared to smaller fish. A more protracted spawning season for large fish is not uncommon and has been observed in albacore tuna [26] and other serial spawners such as anchovies, sardines, pilchards, cods and mackerels (see [30]).

SBT arriving on the spawning ground may require a period of final ovary maturation before spawning commences. Farley and Davis [8] found that pre-spawning SBT south of, and in transit to, the spawning ground had a high incidence of α stage atresia in their ovaries. They concluded that SBT arrive on the spawning ground in this condition or just recovering from it. This is supported by the results of the present study based on a larger sample size, as the proportion of non-spawning fish often with moderate levels of α atresia (10–50%) was highest at the start of the spawning season. Atresia of yolked oocytes can occur any time in species with asynchronous oocyte development [31] and may be a mechanism for fish to reallocate energy from the gonads during unfavourable conditions [32]. Ovarian follicular atresia prior to spawning has been documented in several species [33] and may be a process used by SBT to regulate the supply of yolked oocytes while migrating to the spawning ground, allowing them to respond to changes in energy demands. Once on the spawning ground, females may delay spawning to allow time to recover from the spawning migration [8] and, or, acclimate to the quite different environmental conditions. However, the delay may also be due to the time required by females to find suitable spawning conditions (such as temperature regimes or thermocline depth), specific spawning aggregations, or to engage in some form of pre-spawning behaviour before they start final oocyte maturation before they start final oocyte maturation. Delays in spawning activity have been identified in aggregating coral reef species in response to inappropriate currents or temperatures [34] but have not been recorded in tuna species.

Non-spawning SBT had higher mean condition and gonad indices compared to actively spawning females, which is consistent with these fish being caught prior to commencing spawning. The decrease in mean condition factor over the spawning season suggests that body reserves are depleted over time as energy is directed into egg production. The presence of non-spawning females throughout the spawning season indicates that there may be a relatively constant fraction of fish newly arriving on the spawning ground which is supported by the relatively high mean *Kn* and GI for non-spawning females in the peak spawning months of November to January, after which it declined. However, non-spawners could also be in a resting phases between spawning episodes during the season. Spawning may cease temporarily if conditions such as food availability or water temperatures deteriorate, or to allow fish to recover temporally from periods of consecutive daily spawning. During the non-spawning episodes, females will continue to develop yolked oocytes from the pool of unyolked oocytes increasing the size of the ovary over time, which is also consistent with the higher mean GI observed in non-spawning females relative to spawning females during the peak spawning months.

Once females start a spawning episode, they spawn daily. It is normal for tunas to spawn at about the same time each day—usually in the late evening or early morning [17, 18, 21, 24, 25, 35]. Thus individual fish appear to spawn at intervals of whole days, not fractions of days. Spawning fraction, however, decreased on average with increasing fish size. For larger SBT, the slightly lower spawning fraction is difficult to explain but may be due to these fish spending more time in a pre-spawning condition when first arriving in the spawning ground, or resting longer between spawning episodes, compared to smaller fish. There was no correlation in spawning fraction with length when data were restricted to spawning fish, i.e. they have completed the pre-spawning phase, or a possible resting phase, between spawning episodes.

Very few estimates of potential annual fecundity are available for tuna [3]. In principle, calculating annual fecundity is simple for a mature fish; being the product of batch fecundity and the number of spawning events per season. Batch fecundity was found to increase with fish size in SBT, although it was quite variable making the relationship uncertain. This level of variability has been observed for many tuna species including bigeye [21], yellowfin [25], albacore [26], black skipjack *Euthynnus lineatus* [35] and skipjack [36]. Likely factors contributing to this variability include geographical variation, inter-annual variation, fish condition, and stage of the spawning cycle—some of which may be interrelated. Schaefer [25] reported both geographical and inter-annual variation in batch fecundity of yellowfin tuna. The predicted batch fecundity of a 125 cm fish was reported to vary from 1.454 million eggs in one year to 2.495 million eggs in the next. Hunter et al. [37] found a two-fold variation in batch fecundity of the well-studied northern anchovy *Engraulis mordax* between years.

Because of the high variability in batch fecundity, Schaefer [3] considered that for most species of tuna the reported fecundity estimates were probably inadequate to estimate annual fecundity. In the current study, it was clear that direct estimation was not going to provide a reasonable estimate of batch fecundity as the standard error of the exponent of log (direct batch fecundity) on log (length) was about 1.4. To reduce this to a reasonable level of around 0.25, would require a 25-fold increase in the number of samples to about 900. The situation is slightly better for estimates of hydrated ovary weight, which have been measured more often and show less individual variation, but even so about a 12-fold increase in sample size would be needed to reduce standard errors to the same level. Obtaining ovary weight data before and after spawning is, however, a relatively inexpensive method to determining the relative changes in batch fecundity with fish size.

Estimating the number of spawning events per season as a function of fish size is more difficult. One approach is to estimate the average duration on the spawning ground divided by the average spawning interval. Unfortunately, there is no obvious way of estimating duration on the spawning ground in the absence of position data from electronic tags. While there have been substantial deployments of electronic tags on SBT [7, 38, 39], to date tags have not been recovered from fish that have visited and returned from the spawning ground (but see [9, 40, 41]). There is, however, data for a related species western Atlantic bluefin tuna which spawn in the Gulf of Mexico between April and June [42]. Electronic tagging data showed that three mature Atlantic bluefin tuna (207–268 curved *FL*) remained on the spawning ground in the Gulf of Mexico for an average of 39 ± 11 days and were in an assumed spawning phase for an average of 18 ± 7 days [42]. The relatively short (average) duration on the spawning ground would suggest a possible preliminary entry phase and then an average of two series of consecutive daily spawning cycles, assuming that the number of consecutive daily spawning/non-spawning events was similar to that of the largest SBT in this study. Similar data are available for eastern Atlantic bluefin tuna that spawn in the Mediterranean Sea between May and July [43]. Electronic tagging of 13 bluefin showed that fish remained on the spawning ground around the Balearic Sea for an average of 31 ± 7 days after tagging, and were assumed to be in a spawning phase for an average of 24 ± 4 of those days [43]. The study also suggested that during the spawning phase, the average fish spawned on 80.3% of days, and spawned for 4.5 ± 3.2 consecutive days before ‘resting’ [43] which is consistent with our *indirect* results for SBT. It is clear from these results, and those from the current study, that an individual spawning season for SBT is likely to be only a small fraction of the spawning season of the population. SBT are likely to remain on the spawning ground for only as long as necessary to complete spawning and then leave. There is an indication that the number of days of sequential spawning increases with fish length in SBT—from 3.6 spawning events for a 150 cm fish to 6.9 spawning events for a 190 cm fish. Although the average duration of both parts of the spawning cycle (spawning and non-spawning) as a function of fish size is known, the total number of cycles is still unknown for SBT. As noted previously, there is some indirect evidence that large fish may have a longer spawning season relative to small fish, since females >180 cm were caught over a greater number of months, but individual residency time on the spawning ground remains unknown.

The present study has provided substantial new information on the spawning dynamics of SBT. The greatly increased number of samples examined enabled the refinement of previous estimates of reproductive parameters and investigation of size related trends. The study developed new methods to estimate relative batch fecundity and the duration of spawning and non-spawning episodes as a function of length. The product of relative batch fecundity and relative number of spawning events per spawning season (if known) would provide an estimate of relative annual fecundity as a function of fish size. Since the size distribution of the SBT spawning stock is monitored annually [26], this information could be used in stock assessments to provide a time series of relative annual fecundity which takes into account information on demographic variation in egg production. There are no estimates of residency time on the spawning ground as a function of fish size and it is recommended that electronic tagging studies on adult SBT be undertaken to address this question. A better understanding of the reproductive behaviour and estimates of relative annual fecundity of SBT will be important for monitoring and assessing the stock and evaluating the implications of alternative rebuilding targets and management procedures for this valuable stock.

## Supporting Information

S1 Table Data underlying the findings described in the manuscript. (DOCX)
