# Supplementary material for: Spawning Dynamics and Size Related Trends in Reproductive Parameters of Southern Bluefin Tuna, Thunnus maccoyii
Source: PLoS One. 2015 May 18;10(5):e0125744. doi: 10.1371/journal.pone.0125744 (PMC4436339; doi:10.1371/journal.pone.0125744)
Supplement: S1 Table — (DOCX) [file pone.0125744.s001.docx]

**Table S1. Data underlying the findings described in the manuscript.**

| Fish number | Year | Month | FL (cm) | BW (g) | GW (g) | MAGO stage | POF stage | α stage atresia | β stage atresia | Batch fecundity |
| --- | --- | --- | --- | --- | --- | --- | --- | --- | --- | --- |
| 1 | 1992 | 10 | 179 | 119 | 3128 | 3 | 0 | 3 | 1 | NA |
| 2 | 1992 | 10 | 180 | 100 | 2366 | 3 | NA | 3 | 1 | NA |
| 3 | 1992 | 10 | 186 | NA | 2551 | 4 | 3 | 1 | 0 | NA |
| 4 | 1992 | 10 | 190 | 121 | 3328 | 3 | 0 | 3 | 1 | NA |
| 5 | 1992 | 11 | 172 | 86 | 2128 | 4 | 3 | 1 | 0 | NA |
| 6 | 1992 | 12 | 181 | 111 | 3686 | 3 | 0 | 3 | 1 | NA |
| 7 | 1992 | 12 | NA | NA | 2965 | 4 | 3 | 1 | 0 | NA |
| 8 | 1993 | 1 | 176 | 82 | 3046 | 3 | 2 | 2 | 1 | NA |
| 9 | 1993 | 1 | 179 | 94 | 3838 | 5 | 1 | 1 | 1 | NA |
| 10 | 1993 | 1 | 188 | 136 | 3777 | 4 | 3 | 3 | 1 | NA |
| 11 | 1993 | 1 | 190 | 102 | 2641 | 4 | 3 | 1 | 0 | NA |
| 12 | 1993 | 1 | 191 | 109 | 1708 | 4 | 3 | 1 | 0 | NA |
| 13 | 1993 | 1 | 191 | 123 | 4370 | 4 | 0 | 3 | 1 | NA |
| 14 | 1993 | 1 | NA | NA | 2726 | 3 | 2 | 2 | 0 | NA |
| 15 | 1993 | 1 | NA | NA | 3464 | 3 | 3 | 1 | 0 | NA |
| 16 | 1993 | 1 | NA | NA | 4056 | 4 | 3 | 1 | 0 | NA |
| 17 | 1993 | 2 | 190 | 101 | 3628 | 3 | 0 | 3 | 1 | NA |
| 18 | 1993 | 2 | 190 | 103 | 3523 | 3 | 0 | 1 | 1 | NA |
| 19 | 1993 | 3 | 190 | 106 | 1806 | 3 | 2 | 2 | 0 | NA |
| 20 | 1993 | 3 | 191 | 112 | 2494 | 4 | 3 | 1 | 0 | NA |
| 21 | 1993 | 3 | 191 | 115 | 1984 | 3 | 3 | 2 | 0 | NA |
| 22 | 1993 | 3 | 194 | 124 | 3486 | 4 | 3 | 1 | 0 | NA |
| 23 | 1993 | 3 | 194 | 125 | 2656 | 3 | NA | 3 | 1 | NA |
| 24 | 1993 | 4 | 199 | 120 | 4771 | 3 | 0 | 3 | 1 | NA |
| 25 | 1993 | 4 | NA | NA | 2980 | 3 | 0 | 3 | 1 | NA |
| 26 | 1993 | 8 | 190 | NA | 3906 | 4 | 3 | 1 | 0 | NA |
| 27 | 1993 | 8 | 192 | 129 | 5104 | 3 | 3 | 3 | 1 | NA |
| 28 | 1993 | 8 | 196 | 155 | 3719 | 3 | 2 | 3 | 1 | NA |
| 29 | 1993 | 9 | 184 | 105 | 2412 | 3 | 2 | 1 | 0 | NA |
| 30 | 1993 | 9 | 187 | 87 | 2570 | 3 | 0 | 3 | 1 | NA |
| 31 | 1993 | 9 | 191 | 104 | 2926 | 3 | 0 | 3 | 1 | NA |
| 32 | 1993 | 9 | 195 | 134 | 5828 | 4 | 3 | 1 | 0 | NA |
| 33 | 1993 | 9 | 197 | 139 | 5309 | 3 | 0 | 3 | 1 | NA |
| 34 | 1993 | 9 | NA | NA | 2543 | 3 | 0 | 3 | 1 | NA |
| 35 | 1993 | 9 | NA | NA | 5162 | 3 | 0 | 3 | 1 | NA |
| 36 | 1993 | 9 | NA | NA | 3468 | 3 | 3 | 1 | 0 | NA |
| 37 | 1993 | 9 | NA | NA | 3687 | 4 | 1 | 3 | 1 | NA |
| 38 | 1993 | 10 | 187 | 86 | 3377 | 4 | 3 | 1 | 0 | NA |
| 39 | 1993 | 10 | 187 | 93 | 1069 | 1 | 0 | 5 | 1 | NA |
| 40 | 1993 | 10 | 189 | 98 | 1526 | 4 | 3 | 2 | 0 | NA |
| 41 | 1993 | 10 | 190 | 109 | 2481 | 3 | 1 | 1 | 0 | NA |
| 42 | 1993 | 10 | 191 | 106 | 2438 | 3 | 2 | 1 | 0 | NA |
| 43 | 1993 | 10 | 191 | 116 | 3708 | 4 | 3 | 1 | 0 | NA |
| 44 | 1993 | 10 | 193 | 114 | 2705 | 3 | 2 | 2 | 0 | NA |
| 45 | 1993 | 10 | 195 | 136 | 5210 | 5 | 0 | 1 | 0 | 9635320 |
| 46 | 1993 | 10 | NA | 101 | 2888 | 3 | 2 | 1 | 0 | NA |
| 47 | 1993 | 10 | NA | 110 | 3133 | 3 | 3 | 1 | 0 | NA |
| 48 | 1993 | 10 | NA | 113 | 2839 | 3 | 0 | 3 | 1 | NA |
| 49 | 1993 | 11 | 188 | 100 | 2535 | 4 | 3 | 1 | 0 | NA |
| 50 | 1993 | 11 | NA | NA | 3649 | 3 | 0 | 3 | 1 | NA |
| 51 | 1993 | 11 | NA | NA | 3984 | 3 | 0 | 3 | 1 | NA |
| 52 | 1993 | 11 | NA | NA | NA | 3 | 0 | 4 | 1 | NA |
| 53 | 1993 | 11 | NA | NA | 2747 | 4 | 3 | 1 | 0 | NA |
| 54 | 1993 | 11 | NA | NA | 2286 | 4 | 3 | 1 | 0 | NA |
| 55 | 1993 | 11 | NA | NA | 4267 | 4 | 3 | 1 | 1 | NA |
| 56 | 1993 | 12 | NA | 64 | 1639 | 4 | 3 | 2 | 1 | NA |
| 57 | 1993 | 12 | NA | 77 | 2801 | 3 | 2 | 3 | 1 | NA |
| 58 | 1993 | 12 | NA | 82 | 2996 | 3 | 0 | 3 | 1 | NA |
| 59 | 1993 | 12 | NA | 84 | 2583 | 3 | 0 | 3 | 1 | NA |
| 60 | 1993 | 12 | NA | 93 | 2558 | 3 | 3 | 1 | 0 | NA |
| 61 | 1993 | 12 | NA | 93 | 3816 | 4 | 3 | 2 | 1 | NA |
| 62 | 1993 | 12 | NA | 94 | 1898 | 3 | 2 | 1 | 0 | NA |
| 63 | 1993 | 12 | NA | 96 | 4661 | 3 | 0 | 3 | 1 | NA |
| 64 | 1993 | 12 | NA | 99 | 3767 | 3 | 3 | 2 | 1 | NA |
| 65 | 1993 | 12 | NA | 102 | 3453 | 4 | 2 | 2 | 1 | NA |
| 66 | 1993 | 12 | NA | 112 | 3915 | 4 | 3 | 2 | 0 | NA |
| 67 | 1993 | 12 | NA | 113 | 3770 | 3 | 2 | 2 | 1 | NA |
| 68 | 1993 | 12 | NA | 118 | 3404 | 4 | 3 | 1 | 0 | NA |
| 69 | 1993 | 12 | NA | 120 | 4666 | 3 | NA | 3 | 1 | NA |
| 70 | 1993 | 12 | NA | 129 | 4377 | 3 | 0 | 3 | 1 | NA |
| 71 | 1993 | 12 | NA | NA | 2852 | 3 | 0 | 3 | 1 | NA |
| 72 | 1993 | 12 | NA | NA | 3316 | 3 | 0 | 3 | 1 | NA |
| 73 | 1993 | 12 | NA | NA | 3700 | 3 | 1 | 2 | 1 | NA |
| 74 | 1993 | 12 | NA | NA | 2523 | 3 | 3 | 1 | 0 | NA |
| 75 | 1993 | 12 | NA | NA | 3905 | 3 | 3 | 1 | 0 | NA |
| 76 | 1993 | 12 | NA | NA | 1932 | 3 | 3 | 1 | 0 | NA |
| 77 | 1993 | 12 | NA | NA | 2468 | 3 | 3 | 2 | 1 | NA |
| 78 | 1993 | 12 | NA | NA | 3246 | 3 | 3 | 2 | 1 | NA |
| 79 | 1993 | 12 | NA | NA | 3281 | 4 | 2 | 2 | 1 | NA |
| 80 | 1993 | 12 | NA | NA | 3238 | 4 | 3 | 1 | 0 | NA |
| 81 | 1993 | 12 | NA | NA | 1737 | 4 | 3 | 1 | 0 | NA |
| 82 | 1993 | 12 | NA | NA | 3015 | 4 | 3 | 1 | 0 | NA |
| 83 | 1993 | 12 | NA | NA | 1888 | 4 | 3 | 1 | 1 | NA |
| 84 | 1994 | 1 | NA | 84 | 1687 | 4 | 3 | 1 | 0 | NA |
| 85 | 1994 | 1 | NA | NA | 3936 | 3 | 0 | 3 | 1 | NA |
| 86 | 1994 | 1 | NA | NA | 4135 | 3 | 0 | 3 | 1 | NA |
| 87 | 1994 | 1 | NA | NA | 4416 | 3 | 0 | 3 | 1 | NA |
| 88 | 1994 | 1 | NA | NA | 3524 | 3 | 0 | 3 | 1 | NA |
| 89 | 1994 | 1 | NA | NA | 4644 | 3 | 0 | 3 | 1 | NA |
| 90 | 1994 | 1 | NA | NA | 2574 | 3 | 2 | 1 | 0 | NA |
| 91 | 1994 | 1 | NA | NA | 3757 | 3 | 2 | 1 | 0 | NA |
| 92 | 1994 | 1 | NA | NA | 2630 | 3 | 2 | 2 | 0 | NA |
| 93 | 1994 | 1 | NA | NA | 3111 | 3 | 3 | 1 | 0 | NA |
| 94 | 1994 | 1 | NA | NA | 2490 | 4 | 3 | 1 | 0 | NA |
| 95 | 1994 | 2 | 176 | 83 | 4467 | 3 | 0 | 3 | 1 | NA |
| 96 | 1994 | 2 | 176 | 85 | 2130 | 3 | 2 | 2 | 0 | NA |
| 97 | 1994 | 2 | 179 | 74 | 1370 | 3 | 2 | 1 | 0 | NA |
| 98 | 1994 | 2 | 179 | 87 | 4577 | 4 | 3 | 1 | 0 | NA |
| 99 | 1994 | 2 | 180 | 85 | 1629 | 4 | 3 | 1 | 1 | NA |
| 100 | 1994 | 2 | 185 | 89 | 3303 | 3 | 3 | 1 | 0 | NA |
| 101 | 1994 | 2 | 185 | 89 | 3319 | 3 | 3 | 1 | 0 | NA |
| 102 | 1994 | 2 | 186 | 93 | 3294 | 3 | 2 | 1 | 1 | NA |
| 103 | 1994 | 2 | 187 | 95 | 3078 | 4 | 0 | 3 | 1 | NA |
| 104 | 1994 | 2 | 188 | 82 | 2749 | 3 | 2 | 2 | 0 | NA |
| 105 | 1994 | 2 | 188 | 99 | 2746 | 3 | 3 | 1 | 0 | NA |
| 106 | 1994 | 2 | 188 | 109 | 1689 | 3 | 3 | 3 | 0 | NA |
| 107 | 1994 | 2 | 189 | 97 | 2060 | 3 | 2 | 3 | 0 | NA |
| 108 | 1994 | 2 | 189 | 105 | 3895 | 3 | 3 | 3 | 1 | NA |
| 109 | 1994 | 2 | 190 | 100 | 2425 | 3 | 2 | 1 | 0 | NA |
| 110 | 1994 | 2 | 190 | 102 | 2677 | 3 | 3 | 2 | 1 | NA |
| 111 | 1994 | 2 | 190 | 103 | 3214 | 4 | 3 | 1 | 0 | NA |
| 112 | 1994 | 2 | 190 | 104 | 3622 | 4 | 3 | 1 | 0 | NA |
| 113 | 1994 | 2 | 190 | 108 | 2534 | 3 | 1 | 4 | 1 | NA |
| 114 | 1994 | 2 | 190 | 109 | 3126 | 3 | 1 | 2 | 1 | NA |
| 115 | 1994 | 2 | 190 | 112 | 2636 | 3 | 0 | 3 | 1 | NA |
| 116 | 1994 | 2 | 190 | 119 | 3698 | 3 | 0 | 3 | 1 | NA |
| 117 | 1994 | 2 | 190 | 126 | 4238 | 3 | 3 | 1 | 0 | NA |
| 118 | 1994 | 2 | 191 | 106 | 3372 | 3 | 3 | 1 | 0 | NA |
| 119 | 1994 | 2 | 191 | 110 | 3639 | 3 | 0 | 3 | 1 | NA |
| 120 | 1994 | 2 | 191 | 115 | 3528 | 3 | 2 | 1 | 1 | NA |
| 121 | 1994 | 2 | 191 | 120 | 4910 | 4 | 3 | 1 | 0 | NA |
| 122 | 1994 | 2 | 191 | 121 | 4996 | 3 | 0 | 3 | 1 | NA |
| 123 | 1994 | 2 | 191 | 124 | 4634 | 3 | 2 | 2 | 1 | NA |
| 124 | 1994 | 2 | 192 | 115 | 5195 | 5 | 3 | 2 | 1 | 6950684 |
| 125 | 1994 | 2 | 192 | 124 | 4443 | 3 | NA | 3 | 0 | NA |
| 126 | 1994 | 2 | 192 | 124 | 4350 | 5 | 3 | 1 | 0 | 6336485 |
| 127 | 1994 | 2 | 193 | 117 | 4386 | 5 | 0 | 1 | 0 | 12285432 |
| 128 | 1994 | 2 | 193 | 145 | 4162 | 3 | 0 | 3 | 1 | NA |
| 129 | 1994 | 2 | 194 | 145 | 2693 | 3 | 0 | 3 | 1 | NA |
| 130 | 1994 | 2 | 197 | 134 | 4546 | 3 | 0 | 3 | 1 | NA |
| 131 | 1994 | 2 | NA | 67 | 3313 | 4 | 3 | 2 | 0 | NA |
| 132 | 1994 | 2 | NA | 88 | 4163 | 3 | 0 | 3 | 1 | NA |
| 133 | 1994 | 2 | NA | 96 | 3120 | 3 | 2 | 1 | 0 | NA |
| 134 | 1994 | 2 | NA | 98 | 2851 | 4 | 3 | 3 | 1 | NA |
| 135 | 1994 | 2 | NA | 125 | 4552 | 3 | 3 | 1 | 0 | NA |
| 136 | 1994 | 2 | NA | 140 | 3741 | 3 | 3 | 1 | 0 | NA |
| 137 | 1994 | 2 | NA | NA | 5063 | 3 | 0 | 3 | 1 | NA |
| 138 | 1994 | 2 | NA | NA | 3619 | 3 | 0 | 3 | 1 | NA |
| 139 | 1994 | 2 | NA | NA | 4970 | 3 | 0 | 3 | 1 | NA |
| 140 | 1994 | 2 | NA | NA | 3315 | 3 | 0 | 4 | 1 | NA |
| 141 | 1994 | 2 | NA | NA | 3082 | 3 | 3 | 1 | 0 | NA |
| 142 | 1994 | 2 | NA | NA | 1916 | 3 | 3 | 2 | 1 | NA |
| 143 | 1994 | 2 | NA | NA | 3003 | 4 | 3 | 1 | 0 | NA |
| 144 | 1994 | 2 | NA | NA | 2215 | 4 | 3 | 1 | 0 | NA |
| 145 | 1994 | 2 | NA | NA | 3225 | 4 | 3 | 1 | 0 | NA |
| 146 | 1994 | 2 | NA | NA | 4892 | 5 | 0 | 1 | 0 | NA |
| 147 | 1994 | 2 | NA | NA | 1900 | 5 | 0 | 1 | 0 | NA |
| 148 | 1994 | 2 | NA | NA | 4005 | 5 | 3 | 1 | 0 | NA |
| 149 | 1994 | 3 | 187 | 91 | 3713 | 4 | 3 | 1 | 0 | NA |
| 150 | 1994 | 3 | 188 | 80 | 4209 | 3 | 0 | 1 | 0 | NA |
| 151 | 1994 | 3 | 188 | 97 | 3578 | 5 | 0 | 3 | 1 | NA |
| 152 | 1994 | 3 | 189 | 102 | 3339 | 4 | 3 | 2 | 0 | NA |
| 153 | 1994 | 3 | 189 | NA | 3397 | 5 | 0 | 3 | 1 | 4082846 |
| 154 | 1994 | 3 | 190 | 90 | 5968 | 5 | 1 | 1 | 1 | NA |
| 155 | 1994 | 3 | 190 | 94 | 3464 | 5 | 0 | 1 | 0 | 4888201 |
| 156 | 1994 | 3 | 190 | 97 | 2779 | 3 | 0 | 3 | 1 | NA |
| 157 | 1994 | 3 | 190 | 100 | 3385 | 3 | 0 | 3 | 1 | NA |
| 158 | 1994 | 3 | 190 | 101 | 7355 | 5 | 0 | 1 | 0 | 16732612 |
| 159 | 1994 | 3 | 190 | 101 | 2715 | 5 | 1 | 1 | 0 | NA |
| 160 | 1994 | 3 | 191 | 105 | 2214 | 3 | NA | 3 | 1 | NA |
| 161 | 1994 | 3 | 191 | 109 | 3798 | 3 | 0 | 3 | 1 | NA |
| 162 | 1994 | 3 | 191 | 112 | 4233 | 3 | 3 | 1 | 0 | NA |
| 163 | 1994 | 3 | 191 | 119 | 1914 | 4 | 3 | 1 | 0 | NA |
| 164 | 1994 | 3 | 191 | 120 | 3135 | 3 | 0 | 3 | 1 | NA |
| 165 | 1994 | 3 | 191 | 126 | 3301 | 3 | 3 | 2 | 1 | NA |
| 166 | 1994 | 3 | 193 | 118 | 4336 | 3 | 3 | 2 | 1 | NA |
| 167 | 1994 | 3 | 194 | 111 | 4234 | 5 | 3 | 1 | 0 | 12684124 |
| 168 | 1994 | 3 | 194 | 135 | 3940 | 5 | 3 | 1 | 0 | 8573144 |
| 169 | 1994 | 3 | 196 | 151 | 4312 | 4 | 3 | 3 | 1 | NA |
| 170 | 1994 | 3 | NA | 128 | 6654 | 3 | 2 | 2 | 1 | NA |
| 171 | 1994 | 3 | NA | NA | 2777 | 3 | 0 | 3 | 1 | NA |
| 172 | 1994 | 3 | NA | NA | 3650 | 3 | 0 | 3 | 1 | NA |
| 173 | 1994 | 3 | NA | NA | 2645 | 3 | 0 | 3 | 1 | NA |
| 174 | 1994 | 3 | NA | NA | 3723 | 3 | 0 | 3 | 1 | NA |
| 175 | 1994 | 3 | NA | NA | 4344 | 3 | 0 | 3 | 1 | NA |
| 176 | 1994 | 3 | NA | NA | 2743 | 3 | 0 | 3 | 1 | NA |
| 177 | 1994 | 3 | NA | NA | 4405 | 3 | 0 | 3 | 1 | NA |
| 178 | 1994 | 3 | NA | NA | 3368 | 3 | 0 | 3 | 1 | NA |
| 179 | 1994 | 3 | NA | NA | 2743 | 3 | 2 | 1 | 0 | NA |
| 180 | 1994 | 3 | NA | NA | 3352 | 3 | 2 | 1 | 0 | NA |
| 181 | 1994 | 3 | NA | NA | 4010 | 3 | 2 | 2 | 0 | NA |
| 182 | 1994 | 3 | NA | NA | 2180 | 3 | 3 | 1 | 0 | NA |
| 183 | 1994 | 3 | NA | NA | 3801 | 3 | 3 | 1 | 0 | NA |
| 184 | 1994 | 3 | NA | NA | 3348 | 3 | 3 | 1 | 1 | NA |
| 185 | 1994 | 3 | NA | NA | 3435 | 3 | 3 | 1 | 1 | NA |
| 186 | 1994 | 3 | NA | NA | 4656 | 3 | 3 | 1 | 1 | NA |
| 187 | 1994 | 3 | NA | NA | 3895 | 3 | 3 | 2 | 0 | NA |
| 188 | 1994 | 3 | NA | NA | 2871 | 3 | 3 | 2 | 0 | NA |
| 189 | 1994 | 3 | NA | NA | 3054 | 3 | 3 | 2 | 1 | NA |
| 190 | 1994 | 3 | NA | NA | 5823 | 3 | 3 | 3 | 0 | NA |
| 191 | 1994 | 3 | NA | NA | 3602 | 3 | 3 | 3 | 1 | NA |
| 192 | 1994 | 3 | NA | NA | 3503 | 3 | NA | 3 | 1 | NA |
| 193 | 1994 | 3 | NA | NA | 3051 | 4 | 3 | 1 | 0 | NA |
| 194 | 1994 | 3 | NA | NA | 2879 | 4 | 3 | 1 | 0 | NA |
| 195 | 1994 | 3 | NA | NA | 3586 | 4 | 3 | 2 | 0 | NA |
| 196 | 1994 | 3 | NA | NA | 4236 | 4 | 3 | 2 | 0 | NA |
| 197 | 1994 | 3 | NA | NA | 2149 | 4 | 3 | 2 | 0 | NA |
| 198 | 1994 | 3 | NA | NA | 3900 | 4 | 3 | 2 | 1 | NA |
| 199 | 1994 | 4 | 181 | 93 | 2985 | 3 | 3 | 1 | 0 | NA |
| 200 | 1994 | 4 | 186 | 107 | 3513 | 3 | 3 | 3 | 1 | NA |
| 201 | 1994 | 4 | 189 | 90 | 2022 | 4 | 3 | 1 | 0 | NA |
| 202 | 1994 | 4 | 189 | 100 | 2836 | 3 | 2 | 3 | 1 | NA |
| 203 | 1994 | 4 | 190 | 91 | 2847 | 3 | 0 | 3 | 1 | NA |
| 204 | 1994 | 4 | 190 | 104 | 2556 | 3 | 0 | 3 | 1 | NA |
| 205 | 1994 | 4 | 191 | 117 | 2203 | 5 | 3 | 1 | 0 | 5494066 |
| 206 | 1994 | 4 | NA | NA | NA | 3 | 0 | 3 | 1 | NA |
| 207 | 1994 | 4 | NA | NA | 4029 | 3 | 2 | 2 | 1 | NA |
| 208 | 1994 | 4 | NA | NA | 3038 | 3 | 3 | 1 | 0 | NA |
| 209 | 1994 | 4 | NA | NA | 3176 | 3 | 3 | 1 | 0 | NA |
| 210 | 1994 | 4 | NA | NA | NA | 3 | 3 | 1 | 0 | NA |
| 211 | 1994 | 4 | NA | NA | NA | 3 | 3 | 1 | 0 | NA |
| 212 | 1994 | 4 | NA | NA | 4474 | 3 | 3 | 1 | 1 | NA |
| 213 | 1994 | 4 | NA | NA | NA | 3 | 3 | 2 | 1 | NA |
| 214 | 1994 | 4 | NA | NA | 2619 | 4 | 3 | 1 | 0 | NA |
| 215 | 1994 | 5 | 181 | 130 | 2000 | 3 | 0 | 2 | 1 | NA |
| 216 | 1994 | 7 | NA | NA | 5973 | 3 | 3 | 1 | 0 | NA |
| 217 | 1994 | 8 | NA | NA | 4134 | 3 | 3 | 3 | 1 | NA |
| 218 | 1994 | 9 | 169 | 115 | 2557 | 3 | 0 | 2 | 1 | NA |
| 219 | 1994 | 9 | 172 | 106 | NA | 3 | 3 | 3 | 1 | NA |
| 220 | 1994 | 9 | 174 | 110 | 2470 | 3 | 0 | 3 | 1 | NA |
| 221 | 1994 | 9 | 177 | 102 | 3517 | 4 | 3 | 1 | 0 | NA |
| 222 | 1994 | 9 | 180 | 110 | 4116 | 4 | 3 | 2 | 1 | NA |
| 223 | 1994 | 9 | 180 | 112 | 779 | 4 | 0 | 1 | 0 | NA |
| 224 | 1994 | 9 | 180 | 114 | 2607 | 3 | 3 | 1 | 0 | NA |
| 225 | 1994 | 9 | 180 | 116 | 3053 | 3 | 0 | 1 | 0 | NA |
| 226 | 1994 | 9 | 182 | 104 | 4798 | 3 | 0 | 3 | 1 | NA |
| 227 | 1994 | 9 | 188 | 127 | 3716 | 4 | 3 | 2 | 0 | NA |
| 228 | 1994 | 9 | 192 | 108 | 3796 | 3 | 2 | 1 | 0 | NA |
| 229 | 1994 | 9 | NA | NA | 2187 | 3 | 0 | 3 | 1 | NA |
| 230 | 1994 | 9 | NA | NA | NA | 3 | 0 | 3 | 1 | NA |
| 231 | 1994 | 9 | NA | NA | 4300 | 3 | 0 | 3 | 1 | NA |
| 232 | 1994 | 9 | NA | NA | 2368 | 3 | 0 | 3 | 1 | NA |
| 233 | 1994 | 9 | NA | NA | NA | 3 | 0 | 3 | 1 | NA |
| 234 | 1994 | 9 | NA | NA | NA | 3 | 0 | 3 | 1 | NA |
| 235 | 1994 | 9 | NA | NA | NA | 3 | 0 | 3 | 1 | NA |
| 236 | 1994 | 9 | NA | NA | 1374 | 3 | 0 | 3 | 1 | NA |
| 237 | 1994 | 9 | NA | NA | 2111 | 3 | 0 | 3 | 1 | NA |
| 238 | 1994 | 9 | NA | NA | 3987 | 3 | 0 | 3 | 1 | NA |
| 239 | 1994 | 9 | NA | NA | 2862 | 3 | 0 | 3 | 1 | NA |
| 240 | 1994 | 9 | NA | NA | 2075 | 3 | 0 | 3 | 1 | NA |
| 241 | 1994 | 9 | NA | NA | 2983 | 3 | 3 | 2 | 0 | NA |
| 242 | 1994 | 9 | NA | NA | 3498 | 4 | 0 | 3 | 1 | NA |
| 243 | 1994 | 9 | NA | NA | 1536 | 4 | 3 | 1 | 0 | NA |
| 244 | 1994 | 9 | NA | NA | 3296 | 4 | 3 | 1 | 0 | NA |
| 245 | 1994 | 9 | NA | NA | 3665 | 4 | 3 | 2 | 1 | NA |
| 246 | 1994 | 9 | NA | NA | NA | 5 | 0 | 1 | 0 | NA |
| 247 | 1994 | 9 | NA | NA | 3420 | 5 | 0 | 1 | 0 | NA |
| 248 | 1994 | 9 | NA | NA | 1314 | 5 | 3 | 1 | 0 | NA |
| 249 | 1994 | 9 | NA | NA | 3750 | 5 | 3 | 2 | 1 | NA |
| 250 | 1994 | 10 | 157 | 61 | 1619 | 4 | 0 | 3 | 1 | NA |
| 251 | 1994 | 10 | 167 | 86 | 1168 | 1 | 0 | 5 | 1 | NA |
| 252 | 1994 | 10 | 174 | 88 | 3197 | 3 | 0 | 3 | 1 | NA |
| 253 | 1994 | 10 | 174 | 124 | 1784 | 3 | 3 | 1 | 0 | NA |
| 254 | 1994 | 10 | 175 | 100 | 5985 | 4 | 0 | 1 | 0 | NA |
| 255 | 1994 | 10 | 176 | 113 | 2444 | 3 | 3 | 1 | 0 | NA |
| 256 | 1994 | 10 | 177 | 92 | 2341 | 3 | 2 | 1 | 0 | NA |
| 257 | 1994 | 10 | 178 | 110 | 4445 | 4 | 0 | 1 | 1 | NA |
| 258 | 1994 | 10 | 178 | 114 | 3939 | 3 | 2 | 3 | 1 | NA |
| 259 | 1994 | 10 | 180 | 92 | 1089 | 3 | 0 | 4 | 1 | NA |
| 260 | 1994 | 10 | 180 | 122 | 4869 | 4 | 3 | 1 | 0 | NA |
| 261 | 1994 | 10 | 182 | 84 | 1283 | 3 | 3 | 3 | 1 | NA |
| 262 | 1994 | 10 | 182 | 89 | 2887 | 3 | 0 | 3 | 1 | NA |
| 263 | 1994 | 10 | 183 | 90 | 2651 | 3 | 2 | 2 | 1 | NA |
| 264 | 1994 | 10 | 184 | 122 | 4134 | 5 | 0 | 1 | 0 | NA |
| 265 | 1994 | 10 | 185 | 124 | 3685 | 4 | 3 | 1 | 0 | NA |
| 266 | 1994 | 10 | 185 | 125 | 4449 | 5 | 3 | 1 | 0 | NA |
| 267 | 1994 | 10 | 188 | 90 | 2450 | 3 | 2 | 1 | 0 | NA |
| 268 | 1994 | 10 | 188 | 135 | 3727 | 3 | 2 | 2 | 0 | NA |
| 269 | 1994 | 10 | 189 | 104 | 3000 | 3 | 0 | 3 | 1 | NA |
| 270 | 1994 | 10 | 190 | 90 | 2000 | 3 | 3 | 1 | 0 | NA |
| 271 | 1994 | 10 | 191 | 103 | 2527 | 3 | 0 | 3 | 1 | NA |
| 272 | 1994 | 10 | 192 | 103 | 1356 | 3 | 3 | 1 | 0 | NA |
| 273 | 1994 | 10 | 192 | 113 | 3104 | 3 | 0 | 1 | 0 | NA |
| 274 | 1994 | 10 | 193 | 114 | 1077 | 3 | 3 | 4 | 1 | NA |
| 275 | 1994 | 10 | 193 | 119 | 3015 | 3 | 0 | 3 | 1 | NA |
| 276 | 1994 | 10 | 197 | 125 | 4000 | 3 | 0 | 3 | 1 | NA |
| 277 | 1994 | 10 | NA | 93 | 2720 | 3 | 1 | 1 | 0 | NA |
| 278 | 1994 | 10 | NA | 93 | 4132 | 5 | 3 | 1 | 1 | NA |
| 279 | 1994 | 10 | NA | NA | 1797 | 1 | 0 | 5 | 1 | NA |
| 280 | 1994 | 10 | NA | NA | 2091 | 3 | 0 | 3 | 1 | NA |
| 281 | 1994 | 10 | NA | NA | 3927 | 3 | 0 | 3 | 1 | NA |
| 282 | 1994 | 10 | NA | NA | 1257 | 3 | 1 | 1 | 0 | NA |
| 283 | 1994 | 10 | NA | NA | 3149 | 3 | 3 | 1 | 0 | NA |
| 284 | 1994 | 10 | NA | NA | 3289 | 3 | 3 | 1 | 0 | NA |
| 285 | 1994 | 10 | NA | NA | 3268 | 3 | 3 | 1 | 0 | NA |
| 286 | 1994 | 10 | NA | NA | 3326 | 3 | 3 | 1 | 1 | NA |
| 287 | 1994 | 10 | NA | NA | 5070 | 3 | 3 | 3 | 1 | NA |
| 288 | 1994 | 10 | NA | NA | 3793 | 4 | 0 | 3 | 1 | NA |
| 289 | 1994 | 10 | NA | NA | 4542 | 4 | 3 | 1 | 0 | NA |
| 290 | 1994 | 10 | NA | NA | 4030 | 4 | 3 | 1 | 0 | NA |
| 291 | 1994 | 10 | NA | NA | 2589 | 4 | 3 | 1 | 0 | NA |
| 292 | 1994 | 10 | NA | NA | 2005 | 4 | 3 | 1 | 0 | NA |
| 293 | 1994 | 11 | 147 | 72 | NA | 3 | 0 | 3 | 1 | NA |
| 294 | 1994 | 11 | 155 | 99 | NA | 3 | 0 | 3 | 1 | NA |
| 295 | 1994 | 11 | 156 | 81 | 4978 | 3 | 3 | 2 | 0 | NA |
| 296 | 1994 | 11 | 161 | 80 | 1290 | 5 | 3 | 1 | 1 | 1187246 |
| 297 | 1994 | 11 | 163 | 79 | 1617 | 5 | 3 | 1 | 0 | 1895235 |
| 298 | 1994 | 11 | 165 | 77 | 3045 | 4 | 3 | 1 | 0 | NA |
| 299 | 1994 | 11 | 165 | 83 | 2728 | 4 | 3 | 1 | 0 | NA |
| 300 | 1994 | 11 | 165 | 94 | 1813 | 3 | 2 | 1 | 0 | NA |
| 301 | 1994 | 11 | 166 | 78 | NA | 3 | 0 | 3 | 1 | NA |
| 302 | 1994 | 11 | 166 | 78 | 2972 | 3 | 0 | 3 | 1 | NA |
| 303 | 1994 | 11 | 166 | 104 | 2459 | 3 | 3 | 2 | 1 | NA |
| 304 | 1994 | 11 | 168 | 74 | 1460 | 3 | 2 | 1 | 0 | NA |
| 305 | 1994 | 11 | 168 | 98 | 2658 | 3 | 3 | 1 | 0 | NA |
| 306 | 1994 | 11 | 170 | 84 | NA | 3 | 2 | 2 | 1 | NA |
| 307 | 1994 | 11 | 170 | 95 | 2296 | 3 | 0 | 3 | 1 | NA |
| 308 | 1994 | 11 | 170 | 95 | NA | 3 | 0 | 3 | 1 | NA |
| 309 | 1994 | 11 | 170 | 109 | NA | 3 | 2 | 1 | 0 | NA |
| 310 | 1994 | 11 | 171 | 95 | 1302 | 3 | 0 | 3 | 1 | NA |
| 311 | 1994 | 11 | 174 | 84 | 2436 | 4 | 3 | 1 | 0 | NA |
| 312 | 1994 | 11 | 174 | 133 | 3163 | 3 | 2 | 3 | 1 | NA |
| 313 | 1994 | 11 | 175 | 85 | 4758 | 4 | 0 | 2 | 0 | NA |
| 314 | 1994 | 11 | 175 | 96 | NA | 4 | 0 | 1 | 1 | NA |
| 315 | 1994 | 11 | 175 | 104 | NA | 3 | 3 | 1 | 0 | NA |
| 316 | 1994 | 11 | 175 | 104 | 4421 | 4 | 3 | 1 | 1 | NA |
| 317 | 1994 | 11 | 176 | 86 | 990 | 3 | 3 | 3 | 1 | NA |
| 318 | 1994 | 11 | 177 | 113 | 3160 | 3 | 0 | 3 | 1 | NA |
| 319 | 1994 | 11 | 178 | 115 | 2516 | 3 | 0 | 3 | 1 | NA |
| 320 | 1994 | 11 | 182 | 103 | 4140 | 3 | 0 | 3 | 1 | NA |
| 321 | 1994 | 11 | 182 | 131 | 2102 | 3 | 0 | 3 | 1 | NA |
| 322 | 1994 | 11 | 185 | 109 | 3823 | 3 | 0 | 3 | 1 | NA |
| 323 | 1994 | 11 | 185 | 126 | 4249 | 3 | 0 | 3 | 1 | NA |
| 324 | 1994 | 11 | 186 | 115 | 1742 | 4 | 3 | 1 | 0 | NA |
| 325 | 1994 | 11 | 188 | 122 | 3025 | 3 | 0 | 3 | 1 | NA |
| 326 | 1994 | 11 | 189 | 120 | 4644 | 3 | 0 | 3 | 1 | NA |
| 327 | 1994 | 11 | 192 | 126 | 2793 | 4 | 3 | 1 | 0 | NA |
| 328 | 1994 | 11 | 195 | 123 | 5001 | 5 | 1 | 3 | 1 | NA |
| 329 | 1994 | 11 | 201 | 131 | 3525 | 3 | 0 | 3 | 0 | NA |
| 330 | 1994 | 11 | 203 | 142 | 2351 | 4 | 3 | 1 | 0 | NA |
| 331 | 1994 | 11 | NA | NA | 2297 | 3 | 3 | 2 | 0 | NA |
| 332 | 1994 | 11 | NA | NA | 2025 | 4 | 3 | 1 | 0 | NA |
| 333 | 1994 | 12 | 156 | 81 | 2786 | 3 | 0 | 2 | 1 | NA |
| 334 | 1994 | 12 | 157 | 89 | 4134 | 3 | 0 | 3 | 1 | NA |
| 335 | 1994 | 12 | 160 | 73 | 1143 | 3 | 3 | 4 | 1 | NA |
| 336 | 1994 | 12 | 160 | 96 | 3065 | 3 | 0 | 3 | 1 | NA |
| 337 | 1994 | 12 | 162 | 83 | 3346 | 4 | 0 | 1 | 1 | NA |
| 338 | 1994 | 12 | 162 | 88 | 4023 | 4 | 0 | 3 | 1 | NA |
| 339 | 1994 | 12 | 163 | 83 | 3315 | 4 | 0 | 3 | 1 | NA |
| 340 | 1994 | 12 | 164 | 104 | 3584 | 5 | 3 | 2 | 1 | NA |
| 341 | 1994 | 12 | 165 | 84 | 1614 | 3 | 3 | 1 | 0 | NA |
| 342 | 1994 | 12 | 167 | 85 | NA | 3 | 0 | 3 | 1 | NA |
| 343 | 1994 | 12 | 167 | 88 | 3076 | 3 | 0 | 3 | 1 | NA |
| 344 | 1994 | 12 | 168 | 108 | 2542 | 4 | 3 | 1 | 0 | NA |
| 345 | 1994 | 12 | 169 | 68 | 2185 | 5 | 2 | 1 | 1 | 4651324 |
| 346 | 1994 | 12 | 170 | 88 | 3313 | 3 | 3 | 1 | 0 | NA |
| 347 | 1994 | 12 | 170 | 116 | 4617 | 3 | 3 | 1 | 0 | NA |
| 348 | 1994 | 12 | 171 | 87 | 2951 | 3 | 0 | 3 | 1 | NA |
| 349 | 1994 | 12 | 171 | 89 | 3979 | 3 | 3 | 2 | 0 | NA |
| 350 | 1994 | 12 | 172 | 86 | 1416 | 3 | 2 | 1 | 0 | NA |
| 351 | 1994 | 12 | 172 | 86 | 2104 | 3 | 3 | 1 | 0 | NA |
| 352 | 1994 | 12 | 172 | 126 | 2417 | 3 | 3 | 1 | 0 | NA |
| 353 | 1994 | 12 | 173 | 88 | 2720 | 3 | 3 | 1 | 0 | NA |
| 354 | 1994 | 12 | 174 | 95 | 5113 | 4 | 3 | 1 | 0 | NA |
| 355 | 1994 | 12 | 174 | 101 | 3503 | 4 | 3 | 1 | 1 | NA |
| 356 | 1994 | 12 | 176 | 97 | 3164 | 3 | 3 | 2 | 0 | NA |
| 357 | 1994 | 12 | 176 | 109 | 4382 | 3 | 0 | 2 | 1 | NA |
| 358 | 1994 | 12 | 179 | 109 | 2747 | 3 | 0 | 3 | 1 | NA |
| 359 | 1994 | 12 | 179 | 114 | 2695 | 3 | 3 | 1 | 0 | NA |
| 360 | 1994 | 12 | 179 | 123 | 1375 | 4 | 3 | 1 | 0 | NA |
| 361 | 1994 | 12 | 179 | 123 | 1621 | 5 | 3 | 1 | 0 | 1770677 |
| 362 | 1994 | 12 | 181 | 106 | 2398 | 3 | 3 | 1 | 0 | NA |
| 363 | 1994 | 12 | 182 | 108 | 1729 | 3 | 3 | 1 | 0 | NA |
| 364 | 1994 | 12 | 184 | 110 | 3769 | 3 | 2 | 1 | 1 | NA |
| 365 | 1994 | 12 | 184 | 115 | 3306 | 3 | 0 | 3 | 1 | NA |
| 366 | 1994 | 12 | 184 | 117 | 3320 | 3 | 0 | 3 | 1 | NA |
| 367 | 1994 | 12 | 185 | 116 | 3083 | 3 | 3 | 1 | 1 | NA |
| 368 | 1994 | 12 | 186 | 120 | 2911 | 3 | 0 | 3 | 1 | NA |
| 369 | 1994 | 12 | 187 | 116 | 2773 | 3 | 0 | 2 | 1 | NA |
| 370 | 1994 | 12 | 190 | 124 | NA | 3 | 2 | 1 | 0 | NA |
| 371 | 1994 | 12 | 191 | 126 | 3217 | 3 | 0 | 3 | 1 | NA |
| 372 | 1994 | 12 | 200 | 156 | 4981 | 3 | 0 | 3 | 1 | NA |
| 373 | 1995 | 1 | 155 | 66 | 2604 | 4 | 3 | 1 | 0 | NA |
| 374 | 1995 | 1 | 161 | 120 | 1342 | 3 | 3 | 1 | 1 | NA |
| 375 | 1995 | 1 | 163 | 82 | 2230 | 3 | 2 | 1 | 0 | NA |
| 376 | 1995 | 1 | 163 | 91 | 1999 | 3 | 3 | 1 | 0 | NA |
| 377 | 1995 | 1 | 163 | 99 | 4074 | 4 | 3 | 1 | 0 | NA |
| 378 | 1995 | 1 | 165 | 74 | 4013 | 4 | 0 | 3 | 1 | NA |
| 379 | 1995 | 1 | 165 | 81 | 2661 | 3 | 3 | 1 | 0 | NA |
| 380 | 1995 | 1 | 165 | 89 | 2913 | 3 | 3 | 1 | 0 | NA |
| 381 | 1995 | 1 | 165 | 95 | NA | 3 | 3 | 1 | 0 | NA |
| 382 | 1995 | 1 | 165 | 97 | 3319 | 3 | 3 | 1 | 0 | NA |
| 383 | 1995 | 1 | 165 | 107 | 3792 | 3 | 0 | 3 | 1 | NA |
| 384 | 1995 | 1 | 166 | 81 | 2687 | 4 | 3 | 1 | 0 | NA |
| 385 | 1995 | 1 | 166 | 109 | 3289 | 3 | 2 | 1 | 0 | NA |
| 386 | 1995 | 1 | 167 | 91 | 3785 | 5 | 2 | 1 | 0 | 2863708 |
| 387 | 1995 | 1 | 168 | 85 | 2615 | 3 | 2 | 1 | 0 | NA |
| 388 | 1995 | 1 | 168 | 107 | NA | 4 | 0 | 1 | 0 | NA |
| 389 | 1995 | 1 | 170 | 70 | 1929 | 3 | 3 | 1 | 0 | NA |
| 390 | 1995 | 1 | 171 | 102 | 2372 | 3 | 3 | 1 | 0 | NA |
| 391 | 1995 | 1 | 172 | 89 | 2080 | 4 | 3 | 1 | 1 | NA |
| 392 | 1995 | 1 | 172 | 103 | 4312 | 4 | 3 | 1 | 0 | NA |
| 393 | 1995 | 1 | 172 | 105 | 2832 | 3 | 3 | 3 | 1 | NA |
| 394 | 1995 | 1 | 172 | 128 | 3583 | 4 | 3 | 1 | 0 | NA |
| 395 | 1995 | 1 | 173 | 91 | 3029 | 3 | 0 | 3 | 1 | NA |
| 396 | 1995 | 1 | 173 | 101 | 2995 | 3 | 2 | 1 | 0 | NA |
| 397 | 1995 | 1 | 174 | 94 | 1518 | 3 | 2 | 1 | 0 | NA |
| 398 | 1995 | 1 | 174 | 96 | 5167 | 5 | 0 | 3 | 1 | 3165643 |
| 399 | 1995 | 1 | 174 | 104 | 4222 | 3 | 0 | 3 | 1 | NA |
| 400 | 1995 | 1 | 175 | 106 | 3585 | 5 | 1 | 1 | 0 | NA |
| 401 | 1995 | 1 | 176 | 96 | 3617 | 3 | 0 | 3 | 1 | NA |
| 402 | 1995 | 1 | 176 | 120 | 3603 | 3 | 3 | 2 | 1 | NA |
| 403 | 1995 | 1 | 177 | 105 | 2050 | 4 | 3 | 1 | 0 | NA |
| 404 | 1995 | 1 | 177 | 108 | 3462 | 4 | 0 | 1 | 0 | NA |
| 405 | 1995 | 1 | 177 | 128 | 4328 | 3 | 3 | 2 | 1 | NA |
| 406 | 1995 | 1 | 178 | 105 | 2048 | 3 | 2 | 1 | 0 | NA |
| 407 | 1995 | 1 | 178 | 108 | 3826 | 3 | 0 | 3 | 1 | NA |
| 408 | 1995 | 1 | 178 | 115 | 3829 | 4 | 3 | 1 | 1 | NA |
| 409 | 1995 | 1 | 179 | 100 | 2952 | 3 | 0 | 3 | 1 | NA |
| 410 | 1995 | 1 | 179 | 105 | 3829 | 3 | 0 | 3 | 1 | NA |
| 411 | 1995 | 1 | 180 | 91 | 2179 | 4 | 3 | 1 | 0 | NA |
| 412 | 1995 | 1 | 180 | 100 | NA | 3 | 0 | 3 | 1 | NA |
| 413 | 1995 | 1 | 180 | 134 | 2182 | 4 | 0 | 1 | 0 | NA |
| 414 | 1995 | 1 | 181 | 113 | 5030 | 3 | 0 | 3 | 1 | NA |
| 415 | 1995 | 1 | 185 | 115 | 3525 | 5 | 3 | 3 | 1 | 4414787 |
| 416 | 1995 | 1 | 185 | 145 | 2676 | 3 | 3 | 1 | 0 | NA |
| 417 | 1995 | 1 | 190 | 126 | 5265 | 5 | 1 | 1 | 0 | NA |
| 418 | 1995 | 1 | 195 | 105 | 3809 | 3 | 0 | 3 | 1 | NA |
| 419 | 1995 | 1 | 200 | 156 | NA | 3 | 0 | 3 | 1 | NA |
| 420 | 1995 | 1 | 206 | 130 | 3671 | 3 | 0 | 3 | 1 | NA |
| 421 | 1995 | 2 | 155 | 82 | 3107 | 3 | 0 | 3 | 1 | NA |
| 422 | 1995 | 2 | 161 | 120 | NA | 4 | 3 | 1 | 0 | NA |
| 423 | 1995 | 2 | 165 | 75 | 3876 | 5 | 0 | 3 | 1 | 4683305 |
| 424 | 1995 | 2 | 166 | 102 | 3808 | 5 | 1 | 1 | 0 | NA |
| 425 | 1995 | 2 | 166 | 109 | 1789 | 3 | 3 | 1 | 0 | NA |
| 426 | 1995 | 2 | 166 | NA | 3170 | 3 | 3 | 2 | 1 | NA |
| 427 | 1995 | 2 | 167 | 100 | 4487 | 3 | 3 | 2 | 0 | NA |
| 428 | 1995 | 2 | 168 | 81 | NA | 3 | 0 | 3 | 1 | NA |
| 429 | 1995 | 2 | 170 | 89 | NA | 3 | 3 | 2 | 1 | NA |
| 430 | 1995 | 2 | 170 | 128 | 4457 | 3 | 2 | 2 | 1 | NA |
| 431 | 1995 | 2 | 170 | 132 | 4178 | 5 | 0 | 3 | 1 | 4106655 |
| 432 | 1995 | 2 | 172 | 91 | NA | 4 | 3 | 1 | 0 | NA |
| 433 | 1995 | 2 | 174 | 93 | NA | 3 | 3 | 1 | 0 | NA |
| 434 | 1995 | 2 | 175 | 100 | NA | 3 | 2 | 3 | 1 | NA |
| 435 | 1995 | 2 | 176 | 98 | NA | 4 | 3 | 1 | 1 | NA |
| 436 | 1995 | 2 | 177 | 82 | 4014 | 4 | 0 | 3 | 1 | NA |
| 437 | 1995 | 2 | 177 | 90 | 3208 | 4 | 3 | 1 | 0 | NA |
| 438 | 1995 | 2 | 177 | 105 | NA | 4 | 3 | 1 | 0 | NA |
| 439 | 1995 | 2 | 179 | 86 | 2321 | 4 | 3 | 1 | 0 | NA |
| 440 | 1995 | 2 | 179 | 102 | NA | 5 | 3 | 1 | 0 | NA |
| 441 | 1995 | 2 | 180 | 103 | 1207 | 3 | 0 | 4 | 1 | NA |
| 442 | 1995 | 2 | 182 | 106 | NA | 3 | 0 | 3 | 1 | NA |
| 443 | 1995 | 2 | 183 | 93 | 3252 | 3 | 0 | 3 | 1 | NA |
| 444 | 1995 | 2 | 183 | 111 | NA | 3 | 0 | 3 | 1 | NA |
| 445 | 1995 | 2 | 184 | 107 | NA | 3 | 0 | 3 | 1 | NA |
| 446 | 1995 | 2 | 184 | 115 | 3898 | 3 | 0 | 3 | 1 | NA |
| 447 | 1995 | 2 | 185 | 114 | 3183 | 3 | 2 | 1 | 0 | NA |
| 448 | 1995 | 2 | 185 | 145 | NA | 4 | 3 | 1 | 0 | NA |
| 449 | 1995 | 2 | 188 | 109 | 2843 | 4 | 3 | 1 | 0 | NA |
| 450 | 1995 | 2 | 189 | 122 | NA | 3 | 0 | 3 | 1 | NA |
| 451 | 1995 | 2 | 189 | 124 | NA | 3 | 2 | 1 | 0 | NA |
| 452 | 1995 | 2 | 203 | 201 | 3113 | 3 | 2 | 1 | 0 | NA |
| 453 | 1995 | 3 | 172 | 106 | 4101 | 3 | 0 | 3 | 1 | NA |
| 454 | 1995 | 3 | 190 | 120 | 4231 | 5 | 0 | 3 | 1 | 5010117 |
| 455 | 1995 | 3 | NA | 99 | 2337 | 4 | 3 | 1 | 0 | NA |
| 456 | 1995 | 3 | NA | 101 | 4591 | 5 | 0 | 1 | 0 | 4083499 |
| 457 | 1995 | 3 | NA | 105 | 4487 | 3 | 0 | 3 | 1 | NA |
| 458 | 1995 | 3 | NA | 106 | 4880 | 3 | 0 | 3 | 1 | NA |
| 459 | 1995 | 3 | NA | 107 | 2495 | 4 | 3 | 1 | 0 | NA |
| 460 | 1999 | 11 | 154 | 77 | 3594 | 4 | 3 | 1 | 0 | NA |
| 461 | 1999 | 11 | 159 | 77 | 2892 | 4 | 3 | 1 | 0 | NA |
| 462 | 1999 | 11 | 170 | 79 | 1737 | 3 | 2 | 1 | 1 | NA |
| 463 | 1999 | 11 | 170 | 84 | 4350 | 4 | 0 | 1 | 0 | NA |
| 464 | 1999 | 11 | 171 | 100 | 3922 | 3 | 2 | 1 | 0 | NA |
| 465 | 1999 | 11 | 172 | 90 | 2267 | 3 | 2 | 1 | 0 | NA |
| 466 | 1999 | 11 | 172 | 93 | 2615 | 3 | 2 | 1 | 0 | NA |
| 467 | 1999 | 11 | 173 | 106 | 4101 | 3 | 2 | 1 | 0 | NA |
| 468 | 1999 | 11 | 180 | 107 | 4379 | 4 | 3 | 1 | 0 | NA |
| 469 | 1999 | 11 | 180 | 110 | 2690 | 3 | 2 | 2 | 1 | NA |
| 470 | 1999 | 11 | 180 | 111 | 2854 | 3 | 3 | 1 | 0 | NA |
| 471 | 1999 | 11 | 180 | 117 | 3491 | 3 | 2 | 1 | 1 | NA |
| 472 | 1999 | 11 | 182 | 113 | 2163 | 3 | 2 | 1 | 1 | NA |
| 473 | 1999 | 11 | 185 | 116 | 3290 | 3 | 2 | 1 | 0 | NA |
| 474 | 1999 | 11 | 190 | 122 | 3891 | 3 | 3 | 1 | 1 | NA |
| 475 | 1999 | 11 | 191 | 134 | 3177 | 3 | 2 | 1 | 0 | NA |
| 476 | 1999 | 11 | 192 | 133 | 2493 | 3 | 2 | 2 | 0 | NA |
| 477 | 1999 | 11 | 193 | 127 | 3456 | 3 | 3 | 1 | 0 | NA |
| 478 | 1999 | 11 | 195 | 134 | 3571 | 4 | 3 | 1 | 1 | NA |
| 479 | 1999 | 11 | 200 | 128 | 3942 | 3 | 1 | 1 | 1 | NA |
| 480 | 1999 | 12 | 158 | 73 | 1849 | 3 | 3 | 1 | 0 | NA |
| 481 | 1999 | 12 | 159 | 74 | 2779 | 4 | 0 | 1 | 0 | NA |
| 482 | 1999 | 12 | 160 | 77 | 3003 | 4 | 0 | 1 | 0 | NA |
| 483 | 1999 | 12 | 160 | 79 | 2699 | 3 | 3 | 1 | 0 | NA |
| 484 | 1999 | 12 | 161 | 70 | 1747 | 3 | 3 | 1 | 0 | NA |
| 485 | 1999 | 12 | 162 | 74 | 2340 | 4 | 3 | 1 | 0 | NA |
| 486 | 1999 | 12 | 162 | 81 | 2635 | 3 | 3 | 1 | 0 | NA |
| 487 | 1999 | 12 | 163 | 70 | 2378 | 3 | 2 | 1 | 0 | NA |
| 488 | 1999 | 12 | 163 | 80 | 1468 | 3 | 3 | 1 | 0 | NA |
| 489 | 1999 | 12 | 164 | 77 | 2598 | 3 | 3 | 1 | 0 | NA |
| 490 | 1999 | 12 | 164 | 86 | 2717 | 3 | 2 | 1 | 0 | NA |
| 491 | 1999 | 12 | 165 | 72 | 2850 | 4 | 3 | 1 | 0 | NA |
| 492 | 1999 | 12 | 166 | 86 | 2055 | 3 | 2 | 1 | 0 | NA |
| 493 | 1999 | 12 | 166 | 86 | 3779 | 3 | 3 | 1 | 1 | NA |
| 494 | 1999 | 12 | 167 | 82 | 1663 | 3 | 3 | 1 | 0 | NA |
| 495 | 1999 | 12 | 167 | 84 | 2827 | 3 | 0 | 1 | 0 | NA |
| 496 | 1999 | 12 | 167 | 87 | 2629 | 3 | 1 | 1 | 0 | NA |
| 497 | 1999 | 12 | 168 | 80 | 2027 | 3 | 2 | 1 | 0 | NA |
| 498 | 1999 | 12 | 168 | 84 | 2406 | 3 | 1 | 1 | 0 | NA |
| 499 | 1999 | 12 | 168 | 87 | 1529 | 3 | 2 | 1 | 0 | NA |
| 500 | 1999 | 12 | 169 | 87 | 2270 | 3 | 2 | 1 | 0 | NA |
| 501 | 1999 | 12 | 169 | 88 | 3464 | 4 | 3 | 2 | 0 | NA |
| 502 | 1999 | 12 | 169 | 89 | 2417 | 3 | 3 | 1 | 0 | NA |
| 503 | 1999 | 12 | 169 | 92 | 3338 | 3 | 3 | 1 | 0 | NA |
| 504 | 1999 | 12 | 169 | 96 | 3548 | 3 | 3 | 1 | 0 | NA |
| 505 | 1999 | 12 | 170 | 81 | 2266 | 3 | 0 | 1 | 0 | NA |
| 506 | 1999 | 12 | 170 | 83 | 3574 | 3 | 3 | 1 | 0 | NA |
| 507 | 1999 | 12 | 170 | 84 | 2864 | 4 | 3 | 1 | 0 | NA |
| 508 | 1999 | 12 | 170 | 86 | 3532 | 3 | 2 | 1 | 0 | NA |
| 509 | 1999 | 12 | 170 | 91 | 2097 | 3 | 3 | 1 | 0 | NA |
| 510 | 1999 | 12 | 170 | 91 | 3648 | 3 | 3 | 2 | 1 | NA |
| 511 | 1999 | 12 | 170 | 93 | 4077 | 3 | 0 | 3 | 0 | NA |
| 512 | 1999 | 12 | 170 | 93 | 3303 | 3 | 0 | 3 | 1 | NA |
| 513 | 1999 | 12 | 170 | 93 | 3239 | 3 | 2 | 1 | 0 | NA |
| 514 | 1999 | 12 | 170 | 94 | 4359 | 3 | 0 | 2 | 1 | NA |
| 515 | 1999 | 12 | 170 | 94 | 2934 | 3 | 3 | 1 | 0 | NA |
| 516 | 1999 | 12 | 171 | 79 | 2604 | 4 | 3 | 1 | 0 | NA |
| 517 | 1999 | 12 | 171 | 89 | 2052 | 3 | NA | 1 | 0 | NA |
| 518 | 1999 | 12 | 171 | 100 | 3884 | 3 | 0 | 3 | 1 | NA |
| 519 | 1999 | 12 | 172 | 85 | 1874 | 3 | 3 | 1 | 0 | NA |
| 520 | 1999 | 12 | 172 | 92 | 1200 | 3 | 0 | 4 | 0 | NA |
| 521 | 1999 | 12 | 172 | 92 | 2563 | 3 | 3 | 1 | 1 | NA |
| 522 | 1999 | 12 | 172 | 92 | 4243 | 4 | 3 | 2 | 1 | NA |
| 523 | 1999 | 12 | 172 | 93 | 2988 | 3 | 3 | 1 | 0 | NA |
| 524 | 1999 | 12 | 172 | 93 | 2528 | 3 | 3 | 1 | 0 | NA |
| 525 | 1999 | 12 | 173 | 88 | 2468 | 4 | 3 | 1 | 0 | NA |
| 526 | 1999 | 12 | 173 | 89 | 1862 | 4 | 2 | 1 | 0 | NA |
| 527 | 1999 | 12 | 173 | 89 | 3062 | 4 | 3 | 1 | 0 | NA |
| 528 | 1999 | 12 | 173 | 91 | 3892 | 3 | 3 | 2 | 1 | NA |
| 529 | 1999 | 12 | 173 | 92 | 2745 | 3 | 2 | 1 | 0 | NA |
| 530 | 1999 | 12 | 173 | 93 | 1492 | 3 | 2 | 1 | 0 | NA |
| 531 | 1999 | 12 | 173 | 95 | 2857 | 3 | 3 | 1 | 1 | NA |
| 532 | 1999 | 12 | 173 | 95 | 4190 | 3 | 3 | 2 | 0 | NA |
| 533 | 1999 | 12 | 174 | 88 | 2769 | 3 | 3 | 1 | 0 | NA |
| 534 | 1999 | 12 | 174 | 94 | 3374 | 3 | 2 | 1 | 0 | NA |
| 535 | 1999 | 12 | 174 | 94 | 2780 | 4 | 3 | 1 | 0 | NA |
| 536 | 1999 | 12 | 174 | 95 | 3464 | 3 | 2 | 1 | 0 | NA |
| 537 | 1999 | 12 | 174 | 96 | 2457 | 3 | 1 | 1 | 0 | NA |
| 538 | 1999 | 12 | 175 | 95 | 2827 | 3 | 3 | 2 | 0 | NA |
| 539 | 1999 | 12 | 175 | 96 | 1613 | 3 | 3 | 1 | 0 | NA |
| 540 | 1999 | 12 | 175 | 97 | 2950 | 3 | 3 | 1 | 0 | NA |
| 541 | 1999 | 12 | 175 | 97 | 3220 | 4 | 3 | 1 | 0 | NA |
| 542 | 1999 | 12 | 175 | 100 | 3736 | 3 | 2 | 1 | 0 | NA |
| 543 | 1999 | 12 | 175 | 102 | 3105 | 3 | 2 | 1 | 0 | NA |
| 544 | 1999 | 12 | 176 | 90 | 3203 | 3 | 2 | 1 | 0 | NA |
| 545 | 1999 | 12 | 176 | 97 | 2394 | 3 | 2 | 1 | 0 | NA |
| 546 | 1999 | 12 | 176 | 105 | 3621 | 3 | 0 | 1 | 0 | NA |
| 547 | 1999 | 12 | 177 | 93 | 2861 | 3 | 3 | 2 | 0 | NA |
| 548 | 1999 | 12 | 177 | 97 | 3171 | 3 | 3 | 1 | 0 | NA |
| 549 | 1999 | 12 | 177 | 99 | 3309 | 3 | 3 | 1 | 0 | NA |
| 550 | 1999 | 12 | 177 | 99 | 3845 | 3 | 3 | 1 | 1 | NA |
| 551 | 1999 | 12 | 177 | 116 | 5482 | 3 | 0 | 2 | 1 | NA |
| 552 | 1999 | 12 | 178 | 94 | 3253 | 3 | 2 | 1 | 0 | NA |
| 553 | 1999 | 12 | 178 | 95 | 3617 | 3 | 2 | 1 | 0 | NA |
| 554 | 1999 | 12 | 178 | 96 | 3852 | 3 | 2 | 1 | 0 | NA |
| 555 | 1999 | 12 | 178 | 98 | 2462 | 3 | 1 | 1 | 0 | NA |
| 556 | 1999 | 12 | 178 | 101 | 2552 | 3 | 2 | 1 | 1 | NA |
| 557 | 1999 | 12 | 178 | 101 | 3200 | 4 | 3 | 1 | 1 | NA |
| 558 | 1999 | 12 | 178 | 102 | 3260 | 4 | 3 | 1 | 0 | NA |
| 559 | 1999 | 12 | 178 | 103 | 3404 | 3 | 2 | 1 | 0 | NA |
| 560 | 1999 | 12 | 178 | 105 | 3894 | 3 | 3 | 1 | 0 | NA |
| 561 | 1999 | 12 | 179 | 87 | 2188 | 3 | 2 | 1 | 1 | NA |
| 562 | 1999 | 12 | 179 | 97 | 3930 | 3 | 3 | 1 | 0 | NA |
| 563 | 1999 | 12 | 179 | 98 | 4022 | 3 | 3 | 1 | 0 | NA |
| 564 | 1999 | 12 | 179 | 103 | 2765 | 3 | 0 | 1 | 0 | NA |
| 565 | 1999 | 12 | 179 | 107 | 3495 | 3 | 2 | 1 | 0 | NA |
| 566 | 1999 | 12 | 180 | 89 | 4305 | 3 | 2 | 1 | 1 | NA |
| 567 | 1999 | 12 | 180 | 98 | 3065 | 3 | 3 | 1 | 0 | NA |
| 568 | 1999 | 12 | 180 | 107 | 2835 | 3 | 0 | 1 | 0 | NA |
| 569 | 1999 | 12 | 180 | 107 | 3147 | 3 | 2 | 1 | 0 | NA |
| 570 | 1999 | 12 | 180 | 116 | 4475 | 3 | 0 | 1 | 0 | NA |
| 571 | 1999 | 12 | 180 | 121 | 3571 | 4 | 3 | 1 | 1 | NA |
| 572 | 1999 | 12 | 181 | 106 | 3436 | 3 | 3 | 1 | 0 | NA |
| 573 | 1999 | 12 | 181 | 107 | 4042 | 3 | 0 | 2 | 1 | NA |
| 574 | 1999 | 12 | 182 | 99 | 3848 | 3 | 1 | 1 | 0 | NA |
| 575 | 1999 | 12 | 182 | 106 | 2433 | 3 | 2 | 1 | 0 | NA |
| 576 | 1999 | 12 | 182 | 108 | 3839 | 3 | 0 | 3 | 1 | NA |
| 577 | 1999 | 12 | 182 | 114 | 3578 | 3 | 1 | 1 | 0 | NA |
| 578 | 1999 | 12 | 183 | 105 | 3203 | 3 | 3 | 1 | 0 | NA |
| 579 | 1999 | 12 | 183 | 106 | 3636 | 3 | 2 | 1 | 0 | NA |
| 580 | 1999 | 12 | 183 | 108 | 3877 | 3 | 2 | 1 | 0 | NA |
| 581 | 1999 | 12 | 183 | 109 | 3415 | 4 | 3 | 1 | 0 | NA |
| 582 | 1999 | 12 | 183 | 110 | 2467 | 3 | 2 | 1 | 0 | NA |
| 583 | 1999 | 12 | 184 | 101 | 3062 | 3 | 2 | 2 | 0 | NA |
| 584 | 1999 | 12 | 184 | 111 | 2042 | 3 | 2 | 1 | 0 | NA |
| 585 | 1999 | 12 | 185 | 112 | 4391 | 4 | 3 | 1 | 0 | NA |
| 586 | 1999 | 12 | 185 | 114 | 4393 | 4 | 2 | 1 | 0 | NA |
| 587 | 1999 | 12 | 185 | 118 | 4738 | 3 | 0 | 1 | 0 | NA |
| 588 | 1999 | 12 | 185 | 118 | 4069 | 3 | 0 | 3 | 0 | NA |
| 589 | 1999 | 12 | 186 | 115 | 4736 | 3 | 0 | 3 | 1 | NA |
| 590 | 1999 | 12 | 186 | 119 | 4483 | 3 | 0 | 1 | 0 | NA |
| 591 | 1999 | 12 | 186 | 119 | 4410 | 3 | 0 | 3 | 1 | NA |
| 592 | 1999 | 12 | 187 | 106 | 3114 | 3 | 3 | 1 | 1 | NA |
| 593 | 1999 | 12 | 187 | 116 | 3443 | 4 | 3 | 1 | 0 | NA |
| 594 | 1999 | 12 | 189 | 107 | 4788 | 4 | 3 | 1 | 1 | NA |
| 595 | 1999 | 12 | 190 | 115 | 3654 | 3 | 2 | 1 | 0 | NA |
| 596 | 1999 | 12 | 191 | 113 | 2379 | 3 | 3 | 1 | 0 | NA |
| 597 | 1999 | 12 | 191 | 124 | 2931 | 3 | 2 | 1 | 0 | NA |
| 598 | 1999 | 12 | 194 | 119 | 3431 | 4 | 2 | 1 | 0 | NA |
| 599 | 1999 | 12 | 194 | 138 | 4478 | 3 | 3 | 1 | 0 | NA |
| 600 | 1999 | 12 | 194 | 144 | 3638 | 3 | 2 | 3 | 0 | NA |
| 601 | 1999 | 12 | 199 | 134 | 5072 | 4 | 3 | 1 | 0 | NA |
| 602 | 1999 | 12 | 200 | 127 | 2276 | 3 | 3 | 2 | 0 | NA |
| 603 | 1999 | 12 | 202 | 159 | 4381 | 3 | 2 | 2 | 0 | NA |
| 604 | 2000 | 1 | 157 | 71 | 2282 | 3 | 3 | 1 | 0 | NA |
| 605 | 2000 | 1 | 159 | 75 | 2370 | 3 | 3 | 1 | 1 | NA |
| 606 | 2000 | 1 | 163 | 77 | 3326 | 4 | 2 | 1 | 0 | NA |
| 607 | 2000 | 1 | 164 | 80 | 3205 | 3 | 0 | 1 | 0 | NA |
| 608 | 2000 | 1 | 170 | 90 | 2854 | 3 | 3 | 1 | 0 | NA |
| 609 | 2000 | 1 | 175 | 95 | 3623 | 3 | 3 | 2 | 1 | NA |
| 610 | 2000 | 1 | 176 | 84 | 1532 | 4 | 3 | 1 | 0 | NA |
| 611 | 2000 | 1 | 176 | 87 | 3455 | 3 | 3 | 4 | 1 | NA |
| 612 | 2000 | 1 | 176 | 98 | 3450 | 3 | 3 | 1 | 0 | NA |
| 613 | 2000 | 1 | 176 | 100 | 3725 | 3 | 2 | 1 | 0 | NA |
| 614 | 2000 | 1 | 179 | 94 | 2133 | 3 | 3 | 1 | 0 | NA |
| 615 | 2000 | 1 | 180 | 104 | 3441 | 3 | 2 | 1 | 0 | NA |
| 616 | 2000 | 1 | 180 | 109 | 10915 | 5 | 3 | 1 | 0 | NA |
| 617 | 2000 | 1 | 182 | 102 | 3606 | 3 | 3 | 4 | 1 | NA |
| 618 | 2000 | 1 | 189 | 125 | 4956 | 3 | 0 | 2 | 0 | NA |
| 619 | 2000 | 1 | 191 | 125 | 5927 | 3 | 0 | 3 | 1 | NA |
| 620 | 2000 | 1 | 193 | 134 | 6148 | 3 | 0 | 3 | 0 | NA |
| 621 | 2000 | 1 | 204 | 162 | 6164 | 3 | 3 | 1 | 0 | NA |
| 622 | 2000 | 2 | 161 | 83 | 1962 | 4 | 0 | 1 | 0 | NA |
| 623 | 2000 | 2 | 170 | 97 | 2910 | 3 | 2 | 1 | 1 | NA |
| 624 | 2000 | 2 | 171 | 100 | 4285 | 4 | 0 | 1 | 0 | NA |
| 625 | 2000 | 2 | 176 | 85 | 3520 | 3 | 3 | 1 | 0 | NA |
| 626 | 2000 | 2 | 177 | 110 | 2251 | 4 | 3 | 1 | 0 | NA |
| 627 | 2000 | 2 | 183 | 116 | 3874 | 4 | 3 | 1 | 0 | NA |
| 628 | 2000 | 2 | 187 | 117 | 3482 | 3 | 2 | 1 | 1 | NA |
| 629 | 2000 | 3 | 152 | 57 | 2669 | 3 | 0 | 1 | 0 | NA |
| 630 | 2000 | 3 | 161 | 83 | 3165 | 3 | 3 | 1 | 1 | NA |
| 631 | 2000 | 3 | 170 | 83 | 4720 | 3 | 0 | 3 | 1 | NA |
| 632 | 2000 | 3 | 170 | 93 | 3143 | 3 | 0 | 1 | 0 | NA |
| 633 | 2000 | 3 | 172 | 96 | 3411 | 3 | 2 | 2 | 0 | NA |
| 634 | 2000 | 3 | 173 | 90 | 4976 | 3 | 0 | 3 | 1 | NA |
| 635 | 2000 | 3 | 173 | 96 | 3496 | 3 | 0 | 3 | 1 | NA |
| 636 | 2000 | 3 | 174 | 94 | 4418 | 5 | 0 | 1 | 0 | NA |
| 637 | 2000 | 3 | 174 | 98 | 2764 | 1 | 0 | 5 | 1 | NA |
| 638 | 2000 | 3 | 175 | 88 | 2956 | 3 | 2 | 1 | 0 | NA |
| 639 | 2000 | 3 | 175 | 108 | 3292 | 3 | 3 | 1 | 0 | NA |
| 640 | 2000 | 3 | 177 | 101 | 3524 | 3 | 0 | 3 | 1 | NA |
| 641 | 2000 | 3 | 178 | 106 | 1584 | 3 | 3 | 1 | 0 | NA |
| 642 | 2000 | 3 | 181 | 106 | 3827 | 3 | 2 | 1 | 0 | NA |
| 643 | 2000 | 3 | 181 | 107 | 3574 | 3 | 0 | 1 | 0 | NA |
| 644 | 2000 | 3 | 183 | 121 | 4513 | 3 | 3 | 2 | 0 | NA |
| 645 | 2000 | 3 | 188 | 111 | 2538 | 4 | 0 | 1 | 0 | NA |
| 646 | 2000 | 3 | 189 | 113 | 3594 | 3 | 3 | 1 | 0 | NA |
| 647 | 2000 | 4 | 162 | 78 | 4000 | 4 | 0 | 3 | 1 | NA |
| 648 | 2000 | 4 | 163 | 81 | 2244 | 3 | 3 | 1 | 1 | NA |
| 649 | 2000 | 4 | 172 | 90 | 3432 | 3 | 0 | 1 | 0 | NA |
| 650 | 2000 | 4 | 178 | 102 | 4920 | 5 | 3 | 1 | 0 | 7768382 |
| 651 | 2000 | 4 | 179 | 96 | 4512 | 4 | 0 | 3 | 1 | NA |
| 652 | 2000 | 4 | 179 | 103 | 3408 | 3 | 0 | 3 | 1 | NA |
| 653 | 2000 | 4 | 180 | 109 | 3104 | 4 | 3 | 1 | 0 | NA |
| 654 | 2000 | 4 | 182 | 125 | 3387 | 4 | 2 | 1 | 0 | NA |
| 655 | 2000 | 4 | 183 | 108 | 4663 | 4 | 0 | 1 | 0 | NA |
| 656 | 2000 | 4 | 185 | 106 | 3607 | 3 | 0 | 2 | 0 | NA |
| 657 | 2000 | 4 | 187 | 119 | 3683 | 3 | 0 | 1 | 1 | NA |
| 658 | 2000 | 4 | 188 | 117 | 2766 | 3 | 0 | 3 | 1 | NA |
| 659 | 2000 | 4 | 193 | 124 | 7856 | 5 | 0 | 1 | 0 | NA |
| 660 | 2000 | 4 | 196 | 134 | 4207 | 3 | 3 | 2 | 1 | NA |
| 661 | 2000 | 4 | 198 | 133 | 2250 | 4 | 0 | 1 | 0 | NA |
| 662 | 2000 | 4 | 199 | 140 | 5552 | 3 | 2 | 1 | 1 | NA |
| 663 | 2000 | 5 | 182 | 128 | 5858 | 3 | 2 | 1 | 1 | NA |
| 664 | 2000 | 5 | 188 | 122 | 4151 | 4 | 3 | 1 | 0 | NA |
| 665 | 2000 | 10 | 152 | 60 | 2408 | 3 | 3 | 3 | 1 | NA |
| 666 | 2000 | 10 | 154 | 79 | 1285 | 3 | 2 | 2 | 0 | NA |
| 667 | 2000 | 10 | 156 | 71 | 3895 | 5 | 3 | 1 | 0 | 4075269 |
| 668 | 2000 | 10 | 158 | 69 | 2160 | 3 | 3 | 1 | 1 | NA |
| 669 | 2000 | 10 | 158 | 87 | 2545 | 3 | 2 | 2 | 1 | NA |
| 670 | 2000 | 10 | 159 | 71 | 3921 | 5 | 3 | 1 | 0 | 5958243 |
| 671 | 2000 | 10 | 159 | 77 | 1088 | 3 | 2 | 2 | 0 | NA |
| 672 | 2000 | 10 | 160 | 72 | 1429 | 3 | 1 | 2 | 0 | NA |
| 673 | 2000 | 10 | 160 | 79 | 3580 | 4 | 0 | 3 | 0 | NA |
| 674 | 2000 | 10 | 161 | 73 | 2199 | 3 | 0 | 3 | 1 | NA |
| 675 | 2000 | 10 | 161 | 79 | 2946 | 3 | 3 | 1 | 0 | NA |
| 676 | 2000 | 10 | 161 | 83 | 2763 | 3 | 3 | 3 | 1 | NA |
| 677 | 2000 | 10 | 162 | 79 | 3188 | 3 | 0 | 3 | 1 | NA |
| 678 | 2000 | 10 | 162 | 80 | 2186 | 3 | 2 | 1 | 0 | NA |
| 679 | 2000 | 10 | 163 | 70 | 2570 | 3 | 0 | 3 | 1 | NA |
| 680 | 2000 | 10 | 163 | 85 | 2558 | 3 | 3 | 1 | 0 | NA |
| 681 | 2000 | 10 | 164 | 81 | 2893 | 5 | 3 | 1 | 0 | NA |
| 682 | 2000 | 10 | 164 | 82 | 3608 | 3 | 0 | 3 | 1 | NA |
| 683 | 2000 | 10 | 165 | 84 | 3312 | 4 | 3 | 3 | 1 | NA |
| 684 | 2000 | 10 | 166 | 82 | 2785 | 3 | 2 | 3 | 0 | NA |
| 685 | 2000 | 10 | 166 | 87 | 2580 | 4 | 3 | 1 | 0 | NA |
| 686 | 2000 | 10 | 167 | 81 | 3140 | 4 | 3 | 1 | 0 | NA |
| 687 | 2000 | 10 | 169 | 84 | 2815 | 3 | 3 | 1 | 1 | NA |
| 688 | 2000 | 10 | 169 | 92 | 3187 | 3 | 2 | 1 | 0 | NA |
| 689 | 2000 | 10 | 170 | 83 | 3645 | 3 | 3 | 2 | 1 | NA |
| 690 | 2000 | 10 | 170 | 88 | 3655 | 4 | 3 | 1 | 0 | NA |
| 691 | 2000 | 10 | 170 | 88 | 2735 | 4 | 3 | 2 | 1 | NA |
| 692 | 2000 | 10 | 170 | 91 | 1116 | 3 | 3 | 1 | 0 | NA |
| 693 | 2000 | 10 | 170 | 92 | 2791 | 3 | 3 | 2 | 0 | NA |
| 694 | 2000 | 10 | 171 | 85 | 3051 | 3 | 0 | 3 | 1 | NA |
| 695 | 2000 | 10 | 172 | 90 | 3097 | 3 | 0 | 3 | 1 | NA |
| 696 | 2000 | 10 | 174 | 75 | 3117 | 3 | 3 | 3 | 1 | NA |
| 697 | 2000 | 10 | 174 | 75 | 4540 | 5 | 3 | 2 | 1 | 4833311 |
| 698 | 2000 | 10 | 174 | 90 | 2910 | 4 | 3 | 1 | 1 | NA |
| 699 | 2000 | 10 | 174 | 95 | 2187 | 3 | 0 | 3 | 1 | NA |
| 700 | 2000 | 10 | 174 | 95 | 2954 | 3 | 3 | 1 | 1 | NA |
| 701 | 2000 | 10 | 176 | 98 | 2640 | 4 | 3 | 2 | 0 | NA |
| 702 | 2000 | 10 | 177 | 89 | 1798 | 3 | 3 | 3 | 1 | NA |
| 703 | 2000 | 10 | 177 | 100 | 2468 | 3 | 2 | 2 | 0 | NA |
| 704 | 2000 | 10 | 177 | 100 | 2208 | 3 | 3 | 1 | 1 | NA |
| 705 | 2000 | 10 | 177 | 101 | 2488 | 3 | 3 | 2 | 0 | NA |
| 706 | 2000 | 10 | 178 | 98 | 3294 | 3 | 0 | 3 | 1 | NA |
| 707 | 2000 | 10 | 178 | 100 | 3586 | 3 | 0 | 3 | 1 | NA |
| 708 | 2000 | 10 | 178 | 101 | 1726 | 3 | 3 | 2 | 0 | NA |
| 709 | 2000 | 10 | 178 | 106 | 1680 | 3 | 3 | 1 | 0 | NA |
| 710 | 2000 | 10 | 178 | 108 | 4372 | 3 | 0 | 3 | 1 | NA |
| 711 | 2000 | 10 | 179 | 103 | 1671 | 3 | 2 | 1 | 1 | NA |
| 712 | 2000 | 10 | 180 | 102 | 3750 | 3 | 0 | 3 | 1 | NA |
| 713 | 2000 | 10 | 180 | 107 | 4899 | 4 | 3 | 1 | 1 | NA |
| 714 | 2000 | 10 | 180 | 108 | 3971 | 3 | 3 | 2 | 1 | NA |
| 715 | 2000 | 10 | 180 | 109 | 4065 | 3 | 0 | 3 | 1 | NA |
| 716 | 2000 | 10 | 181 | 106 | 3838 | 5 | 3 | 1 | 0 | NA |
| 717 | 2000 | 10 | 181 | 108 | 2830 | 4 | NA | 1 | 1 | NA |
| 718 | 2000 | 10 | 181 | 116 | 4613 | 3 | 3 | 3 | 1 | NA |
| 719 | 2000 | 10 | 182 | 100 | 4125 | 3 | 3 | 3 | 1 | NA |
| 720 | 2000 | 10 | 182 | 106 | 3127 | 3 | 3 | 2 | 1 | NA |
| 721 | 2000 | 10 | 182 | 106 | 4329 | 4 | 2 | 1 | 0 | NA |
| 722 | 2000 | 10 | 183 | 109 | 3265 | 3 | 3 | 2 | 1 | NA |
| 723 | 2000 | 10 | 183 | 111 | 3045 | 3 | 3 | 1 | 1 | NA |
| 724 | 2000 | 10 | 183 | 115 | 3247 | 3 | 3 | 3 | 1 | NA |
| 725 | 2000 | 10 | 184 | 118 | 4433 | 3 | 0 | 3 | 1 | NA |
| 726 | 2000 | 10 | 185 | 121 | 4965 | 3 | 3 | 3 | 1 | NA |
| 727 | 2000 | 10 | 187 | 110 | 1426 | 1 | 0 | 5 | 1 | NA |
| 728 | 2000 | 10 | 188 | 121 | 3238 | 3 | 3 | 2 | 1 | NA |
| 729 | 2000 | 10 | 195 | 131 | 3672 | 3 | 3 | 2 | 0 | NA |
| 730 | 2000 | 10 | 195 | 131 | 5198 | 5 | 3 | 1 | 1 | 10384086 |
| 731 | 2000 | 11 | 157 | 75 | 2286 | 3 | 3 | 1 | 0 | NA |
| 732 | 2000 | 11 | 161 | 81 | 3428 | 3 | 2 | 1 | 1 | NA |
| 733 | 2000 | 11 | 161 | 85 | 3820 | 5 | 0 | 1 | 0 | NA |
| 734 | 2000 | 11 | 162 | 82 | 2188 | 3 | 3 | 1 | 0 | NA |
| 735 | 2000 | 11 | 162 | 83 | 3720 | 3 | 0 | 2 | 0 | NA |
| 736 | 2000 | 11 | 164 | 72 | 2244 | 4 | 3 | 1 | 0 | NA |
| 737 | 2000 | 11 | 168 | 87 | 3018 | 4 | 3 | 1 | 0 | NA |
| 738 | 2000 | 11 | 168 | 88 | 3488 | 4 | 3 | 2 | 0 | NA |
| 739 | 2000 | 11 | 170 | 87 | 5886 | 5 | NA | NA | NA | 11602409 |
| 740 | 2000 | 11 | 172 | 82 | 4279 | 3 | 0 | 3 | 1 | NA |
| 741 | 2000 | 11 | 172 | 90 | 3910 | 3 | 2 | 3 | 1 | NA |
| 742 | 2000 | 11 | 173 | 94 | 2082 | 3 | 3 | 1 | 1 | NA |
| 743 | 2000 | 11 | 175 | 92 | 3348 | 3 | 2 | 3 | 1 | NA |
| 744 | 2000 | 11 | 175 | 96 | 3852 | 3 | 0 | 3 | 1 | NA |
| 745 | 2000 | 11 | 176 | 94 | 3598 | 3 | 2 | 1 | 1 | NA |
| 746 | 2000 | 11 | 176 | 94 | 3145 | 3 | 3 | 1 | 0 | NA |
| 747 | 2000 | 11 | 176 | 100 | 2035 | 4 | 3 | 3 | 1 | NA |
| 748 | 2000 | 11 | 177 | 89 | 2605 | 3 | 3 | 1 | 1 | NA |
| 749 | 2000 | 11 | 178 | 100 | 2614 | 3 | 3 | 1 | 0 | NA |
| 750 | 2000 | 11 | 179 | 117 | 2948 | 3 | 3 | 1 | 0 | NA |
| 751 | 2000 | 11 | 180 | 100 | 3871 | 3 | 3 | 2 | 1 | NA |
| 752 | 2000 | 11 | 180 | 103 | 3450 | 3 | 0 | 1 | 0 | NA |
| 753 | 2000 | 11 | 180 | 113 | 4288 | 4 | 3 | 1 | 1 | NA |
| 754 | 2000 | 11 | 181 | 119 | 5796 | 4 | 3 | 3 | 1 | NA |
| 755 | 2000 | 11 | 185 | 107 | 4456 | 4 | 3 | 3 | 1 | NA |
| 756 | 2000 | 11 | 185 | 111 | 3745 | 3 | 0 | 2 | 1 | NA |
| 757 | 2000 | 11 | 187 | 101 | 3054 | 3 | 0 | 2 | 1 | NA |
| 758 | 2000 | 11 | 188 | 121 | 6285 | 5 | 0 | 1 | 0 | NA |
| 759 | 2000 | 11 | 189 | 108 | 2945 | 3 | 1 | 1 | 0 | NA |
| 760 | 2000 | 11 | 189 | 117 | 5145 | 3 | 3 | 1 | 0 | NA |
| 761 | 2000 | 11 | 190 | 111 | 1868 | 5 | 3 | 3 | 1 | NA |
| 762 | 2000 | 11 | 190 | 116 | 3986 | 3 | 2 | 1 | 1 | NA |
| 763 | 2000 | 11 | 190 | 125 | 3278 | 4 | 3 | 1 | 1 | NA |
| 764 | 2000 | 11 | 194 | 128 | 4956 | 3 | 3 | 1 | 1 | NA |
| 765 | 2000 | 11 | 199 | 138 | 4955 | 3 | 3 | 3 | 1 | NA |
| 766 | 2000 | 11 | 210 | 170 | 5856 | 3 | 0 | 3 | 1 | NA |
| 767 | 2000 | 12 | 168 | 97 | 4851 | 4 | 3 | 3 | 1 | NA |
| 768 | 2000 | 12 | 170 | 90 | 6063 | 3 | 3 | 1 | 0 | NA |
| 769 | 2000 | 12 | 175 | 94 | 3264 | 3 | 3 | 3 | 1 | NA |
| 770 | 2000 | 12 | 176 | 97 | 3257 | 3 | 3 | 3 | 1 | NA |
| 771 | 2000 | 12 | 178 | 100 | 3016 | 3 | 3 | 2 | 1 | NA |
| 772 | 2000 | 12 | 181 | 120 | 1723 | 4 | 3 | 2 | 1 | NA |
| 773 | 2001 | 1 | 154 | 63 | 2339 | 4 | 3 | 1 | 0 | NA |
| 774 | 2001 | 1 | 158 | 69 | 2707 | 3 | 3 | 3 | 1 | NA |
| 775 | 2001 | 1 | 159 | 72 | 2886 | 4 | 3 | 1 | 0 | NA |
| 776 | 2001 | 1 | 160 | 76 | 2682 | 3 | 3 | 1 | 0 | NA |
| 777 | 2001 | 1 | 161 | 69 | 2098 | 3 | 3 | 1 | 0 | NA |
| 778 | 2001 | 1 | 161 | 80 | 2594 | 3 | 3 | 1 | 0 | NA |
| 779 | 2001 | 1 | 162 | 76 | 2204 | 3 | 3 | 2 | 1 | NA |
| 780 | 2001 | 1 | 162 | 81 | 2742 | 3 | 3 | 1 | 1 | NA |
| 781 | 2001 | 1 | 162 | 86 | 7490 | 3 | 3 | 1 | 1 | NA |
| 782 | 2001 | 1 | 162 | 93 | 4258 | 3 | 0 | 3 | 1 | NA |
| 783 | 2001 | 1 | 165 | 75 | 2406 | 3 | 2 | 1 | 0 | NA |
| 784 | 2001 | 1 | 166 | 90 | 3387 | 3 | 3 | 2 | 0 | NA |
| 785 | 2001 | 1 | 167 | 80 | 6488 | 5 | 1 | 1 | 0 | 13620566 |
| 786 | 2001 | 1 | 167 | 81 | 3906 | 3 | 2 | 1 | 0 | NA |
| 787 | 2001 | 1 | 168 | 81 | 2528 | 3 | 2 | 1 | 0 | NA |
| 788 | 2001 | 1 | 169 | 80 | 2756 | 3 | 2 | 1 | 0 | NA |
| 789 | 2001 | 1 | 170 | 79 | 1289 | 3 | 3 | 1 | 0 | NA |
| 790 | 2001 | 1 | 170 | 87 | 2598 | 3 | 2 | 1 | 0 | NA |
| 791 | 2001 | 1 | 170 | 87 | 1216 | 3 | 3 | 1 | 1 | NA |
| 792 | 2001 | 1 | 171 | 84 | 1765 | 4 | 3 | 2 | 0 | NA |
| 793 | 2001 | 1 | 171 | 85 | 2432 | 4 | 3 | 1 | 1 | NA |
| 794 | 2001 | 1 | 172 | 88 | 3388 | 4 | 3 | 1 | 0 | NA |
| 795 | 2001 | 1 | 172 | 89 | 3290 | 3 | 3 | 1 | 1 | NA |
| 796 | 2001 | 1 | 175 | 96 | 2346 | 3 | 3 | 2 | 0 | NA |
| 797 | 2001 | 1 | 177 | 99 | 3729 | 3 | 3 | 3 | 0 | NA |
| 798 | 2001 | 1 | 178 | 103 | 3450 | 3 | 3 | 1 | 1 | NA |
| 799 | 2001 | 1 | 178 | 104 | 3658 | 3 | 3 | 2 | 1 | NA |
| 800 | 2001 | 1 | 180 | 104 | 3480 | 3 | 3 | 1 | 0 | NA |
| 801 | 2001 | 1 | 180 | 104 | 2759 | 4 | 3 | 1 | 0 | NA |
| 802 | 2001 | 1 | 181 | 126 | 5426 | 4 | 3 | 1 | 0 | NA |
| 803 | 2001 | 1 | 183 | 110 | 3506 | 3 | 2 | 3 | 1 | NA |
| 804 | 2001 | 1 | 185 | 115 | 4088 | 3 | 0 | 3 | 1 | NA |
| 805 | 2001 | 1 | 187 | 126 | 4708 | 3 | 3 | 3 | 1 | NA |
| 806 | 2001 | 1 | 188 | 128 | 7240 | 5 | 3 | 2 | 1 | 8489605 |
| 807 | 2001 | 1 | 189 | 106 | 1798 | 4 | 3 | 1 | 0 | NA |
| 808 | 2001 | 1 | 192 | 130 | 3098 | 3 | 2 | 1 | 0 | NA |
| 809 | 2001 | 2 | 158 | 76 | 2962 | 3 | 2 | 1 | 0 | NA |
| 810 | 2001 | 2 | 168 | 86 | 2510 | 3 | 3 | 1 | 0 | NA |
| 811 | 2001 | 2 | 169 | 87 | 2289 | 3 | 0 | 3 | 1 | NA |
| 812 | 2001 | 2 | 170 | 92 | 2607 | 3 | 2 | 1 | 0 | NA |
| 813 | 2001 | 2 | 170 | 93 | 3157 | 3 | 3 | 1 | 0 | NA |
| 814 | 2001 | 2 | 178 | 99 | 3310 | 3 | 3 | 1 | 1 | NA |
| 815 | 2001 | 2 | 178 | 100 | 3329 | 3 | 3 | 1 | 1 | NA |
| 816 | 2001 | 2 | 182 | 106 | 5201 | 4 | 3 | 1 | 1 | NA |
| 817 | 2001 | 2 | 182 | 110 | 3188 | 3 | 3 | 1 | 0 | NA |
| 818 | 2001 | 2 | 182 | 112 | 2922 | 3 | 3 | 2 | 1 | NA |
| 819 | 2001 | 2 | 184 | 116 | 4681 | 3 | 3 | 2 | 1 | NA |
| 820 | 2001 | 2 | 186 | 117 | 2530 | 4 | 3 | 1 | 1 | NA |
| 821 | 2001 | 2 | 186 | 118 | 4442 | 3 | 0 | 3 | 1 | NA |
| 822 | 2001 | 2 | 187 | 115 | 4856 | 4 | 3 | 1 | 0 | NA |
| 823 | 2001 | 2 | 187 | 119 | 2564 | 3 | 0 | 3 | 1 | NA |
| 824 | 2001 | 2 | 189 | 126 | 5986 | 3 | 0 | 3 | 1 | NA |
| 825 | 2001 | 3 | 175 | 97 | 2486 | 4 | 3 | 2 | 1 | NA |
| 826 | 2001 | 3 | 176 | 99 | 3089 | 3 | 2 | 2 | 1 | NA |
| 827 | 2001 | 3 | 178 | 108 | 6280 | 4 | 3 | 2 | 1 | NA |
| 828 | 2001 | 3 | 182 | 108 | 3214 | 3 | 3 | 2 | 1 | NA |
| 829 | 2001 | 3 | 182 | 130 | 7250 | 4 | 3 | 2 | 1 | NA |
| 830 | 2001 | 3 | 184 | 112 | 3598 | 4 | 3 | 3 | 1 | NA |
| 831 | 2001 | 3 | 185 | 117 | 4284 | 3 | 0 | 3 | 1 | NA |
| 832 | 2001 | 9 | 172 | 95 | 1561 | 3 | 0 | 2 | 1 | NA |
| 833 | 2001 | 9 | 182 | 117 | 2188 | 3 | 0 | 3 | 1 | NA |
| 834 | 2001 | 9 | 191 | 134 | 3020 | 3 | 0 | 3 | 1 | NA |
| 835 | 2001 | 10 | 155 | 69 | 870 | 3 | 0 | 2 | 1 | NA |
| 836 | 2001 | 10 | 155 | 71 | 1716 | 4 | 3 | 2 | 0 | NA |
| 837 | 2001 | 10 | 155 | 73 | 562 | 3 | 0 | 2 | 0 | NA |
| 838 | 2001 | 10 | 155 | 75 | 1862 | 3 | 2 | 2 | 0 | NA |
| 839 | 2001 | 10 | 156 | 72 | 1905 | 3 | 2 | 1 | 0 | NA |
| 840 | 2001 | 10 | 156 | 73 | 659 | 3 | 0 | 1 | 0 | NA |
| 841 | 2001 | 10 | 156 | 77 | 1788 | 3 | 2 | 1 | 0 | NA |
| 842 | 2001 | 10 | 157 | 74 | 1458 | 3 | 3 | 3 | 1 | NA |
| 843 | 2001 | 10 | 157 | 75 | 717 | 4 | 1 | 1 | 0 | NA |
| 844 | 2001 | 10 | 158 | 80 | 2112 | 3 | 2 | 1 | 0 | NA |
| 845 | 2001 | 10 | 158 | 80 | 1090 | 3 | 2 | 2 | 1 | NA |
| 846 | 2001 | 10 | 159 | 75 | 1625 | 3 | 3 | 3 | 1 | NA |
| 847 | 2001 | 10 | 160 | 82 | 1244 | 3 | 2 | 1 | 0 | NA |
| 848 | 2001 | 10 | 160 | 82 | 1925 | 3 | 2 | 2 | 0 | NA |
| 849 | 2001 | 10 | 160 | 83 | 1674 | 3 | 0 | 3 | 1 | NA |
| 850 | 2001 | 10 | 162 | 85 | 2526 | 4 | 3 | 1 | 0 | NA |
| 851 | 2001 | 10 | 163 | 86 | 1547 | 3 | 3 | 1 | 0 | NA |
| 852 | 2001 | 10 | 163 | 88 | 1886 | 3 | 2 | 1 | 0 | NA |
| 853 | 2001 | 10 | 164 | 84 | 2511 | 3 | 2 | 1 | 0 | NA |
| 854 | 2001 | 10 | 164 | 86 | 1865 | 3 | 3 | 2 | 1 | NA |
| 855 | 2001 | 10 | 164 | 88 | 1015 | 3 | 3 | 1 | 0 | NA |
| 856 | 2001 | 10 | 165 | 88 | 2291 | 3 | 3 | 1 | 1 | NA |
| 857 | 2001 | 10 | 168 | 90 | 2161 | 3 | 3 | 1 | 0 | NA |
| 858 | 2001 | 10 | 168 | 91 | 1701 | 3 | 3 | 1 | 1 | NA |
| 859 | 2001 | 10 | 168 | 92 | 1390 | 3 | 2 | 1 | 0 | NA |
| 860 | 2001 | 10 | 170 | 90 | 2484 | 3 | 3 | 2 | 1 | NA |
| 861 | 2001 | 10 | 170 | 92 | 1829 | 3 | 3 | 1 | 0 | NA |
| 862 | 2001 | 10 | 170 | 92 | 1605 | 3 | 3 | 1 | 1 | NA |
| 863 | 2001 | 10 | 170 | 93 | 1734 | 3 | 2 | 1 |  | NA |
| 864 | 2001 | 10 | 170 | 93 | 1886 | 3 | 3 | 1 | 0 | NA |
| 865 | 2001 | 10 | 170 | 94 | 3380 | 3 | 0 | 3 | 1 | NA |
| 866 | 2001 | 10 | 170 | 97 | 2239 | 3 | 2 | 1 | 0 | NA |
| 867 | 2001 | 10 | 171 | 94 | 2126 | 3 | 3 | 1 | 0 | NA |
| 868 | 2001 | 10 | 172 | 94 | 1672 | 4 | 3 | 1 | 0 | NA |
| 869 | 2001 | 10 | 172 | 95 | 1765 | 3 | 0 | 3 | 1 | NA |
| 870 | 2001 | 10 | 172 | 96 | 3725 | 3 | 0 | 3 | 1 | NA |
| 871 | 2001 | 10 | 172 | 96 | 2360 | 3 | 2 | 1 | 0 | NA |
| 872 | 2001 | 10 | 172 | 97 | 2136 | 3 | 3 | 1 | 1 | NA |
| 873 | 2001 | 10 | 175 | 96 | 4477 | 3 | 0 | 3 | 1 | NA |
| 874 | 2001 | 10 | 175 | 97 | 2354 | 3 | 3 | 1 | 1 | NA |
| 875 | 2001 | 10 | 175 | 100 | 2822 | 3 | 0 | 3 | 1 | NA |
| 876 | 2001 | 10 | 175 | 104 | 2918 | 3 | 3 | 3 | 1 | NA |
| 877 | 2001 | 10 | 176 | 101 | 5122 | 3 | 3 | 3 | 1 | NA |
| 878 | 2001 | 10 | 176 | 102 | 2311 | 3 | 1 | 2 | 1 | NA |
| 879 | 2001 | 10 | 178 | 98 | 2724 | 3 | 0 | 3 | 1 | NA |
| 880 | 2001 | 10 | 178 | 100 | 4284 | 3 | 0 | 3 | 1 | NA |
| 881 | 2001 | 10 | 178 | 100 | 2427 | 3 | 3 | 1 | 0 | NA |
| 882 | 2001 | 10 | 178 | 104 | 2380 | 4 | 3 | 1 | 1 | NA |
| 883 | 2001 | 10 | 179 | 106 | 2256 | 5 | 3 | 1 | 0 | 3496633 |
| 884 | 2001 | 10 | 180 | 106 | 3101 | 3 | 2 | 3 | 1 | NA |
| 885 | 2001 | 10 | 181 | 106 | 4357 | 4 | 3 | 1 | 0 | NA |
| 886 | 2001 | 10 | 181 | 108 | 2712 | 4 | 0 | 3 | 1 | NA |
| 887 | 2001 | 10 | 181 | 109 | 2781 | 3 | 3 | 1 | 0 | NA |
| 888 | 2001 | 10 | 181 | 109 | 2351 | 3 | 3 | 1 | 0 | NA |
| 889 | 2001 | 10 | 181 | 110 | 3372 | 3 | 3 | 3 | 1 | NA |
| 890 | 2001 | 10 | 182 | 111 | 2313 | 3 | 2 | 2 | 1 | NA |
| 891 | 2001 | 10 | 182 | 112 | 2306 | 3 | 2 | 2 | 1 | NA |
| 892 | 2001 | 10 | 182 | 112 | 2736 | 3 | 3 | 2 | 1 | NA |
| 893 | 2001 | 10 | 182 | 114 | 2282 | 4 | 0 | 1 | 0 | NA |
| 894 | 2001 | 10 | 182 | 116 | 3025 | 3 | 0 | 4 | 1 | NA |
| 895 | 2001 | 10 | 182 | 117 | 3156 | 3 | 3 | 1 | 0 | NA |
| 896 | 2001 | 10 | 183 | 112 | 2373 | 3 | 2 | 2 | 1 | NA |
| 897 | 2001 | 10 | 184 | 114 | 2976 | 3 | 2 | 1 | 0 | NA |
| 898 | 2001 | 10 | 184 | 118 | 2614 | 5 | 3 | 1 | 0 | 5303656 |
| 899 | 2001 | 10 | 184 | 119 | 3892 | 4 | 0 | 3 | 1 | NA |
| 900 | 2001 | 10 | 185 | 118 | 3744 | 3 | 0 | 3 | 1 | NA |
| 901 | 2001 | 10 | 185 | 124 | 4147 | 3 | 0 | 3 | 1 | NA |
| 902 | 2001 | 10 | 186 | 120 | 3430 | 3 | 0 | 3 | 1 | NA |
| 903 | 2001 | 10 | 186 | 121 | 3284 | 3 | 3 | 3 | 1 | NA |
| 904 | 2001 | 10 | 186 | 124 | 2964 | 3 | 3 | 2 | 1 | NA |
| 905 | 2001 | 10 | 186 | 128 | 4366 | 3 | 0 | 3 | 1 | NA |
| 906 | 2001 | 10 | 189 | 127 | 4276 | 3 | 0 | 3 | 1 | NA |
| 907 | 2001 | 10 | 189 | 128 | 2917 | 3 | 3 | 2 | 1 | NA |
| 908 | 2001 | 10 | 189 | 132 | 3798 | 4 | 0 | 3 | 1 | NA |
| 909 | 2001 | 10 | 189 | 133 | 2621 | 3 | 2 | 1 | 0 | NA |
| 910 | 2001 | 10 | 190 | 138 | 5020 | 3 | 0 | 3 | 1 | NA |
| 911 | 2001 | 10 | 199 | 144 | 5266 | 3 | 0 | 3 | 1 | NA |
| 912 | 2001 | 10 | 208 | 151 | 5025 | 3 | 0 | 3 | 1 | NA |
| 913 | 2001 | 11 | 141 | 73 | 1865 | 3 | 0 | 3 | 1 | NA |
| 914 | 2001 | 11 | 143 | 74 | 770 | 4 | 3 | 1 | 1 | NA |
| 915 | 2001 | 11 | 146 | 81 | 2232 | 3 | 0 | 3 | 1 | NA |
| 916 | 2001 | 11 | 147 | 75 | 1648 | 3 | 0 | 3 | 1 | NA |
| 917 | 2001 | 11 | 150 | 84 | 1195 | 3 | 0 | 4 | 1 | NA |
| 918 | 2001 | 11 | 152 | 71 | 2378 | 3 | 0 | 3 | 1 | NA |
| 919 | 2001 | 11 | 155 | 81 | 2361 | 3 | 0 | 3 | 1 | NA |
| 920 | 2001 | 11 | 155 | 85 | 2520 | 3 | 0 | 3 | 1 | NA |
| 921 | 2001 | 11 | 156 | 71 | 1581 | 3 | 2 | 1 | 0 | NA |
| 922 | 2001 | 11 | 157 | 109 | 2358 | 3 | 3 | 1 | 0 | NA |
| 923 | 2001 | 11 | 160 | 88 | 2270 | 3 | 3 | 1 | 0 | NA |
| 924 | 2001 | 11 | 160 | 92 | 1785 | 3 | 0 | 3 | 1 | NA |
| 925 | 2001 | 11 | 161 | 70 | 1743 | 3 | 3 | 1 | 0 | NA |
| 926 | 2001 | 11 | 161 | 74 | 1675 | 5 | 3 | 1 | 0 | NA |
| 927 | 2001 | 11 | 162 | 87 | 2075 | 3 | 3 | 1 | 0 | NA |
| 928 | 2001 | 11 | 163 | 80 | 1508 | 3 | 2 | 1 | 0 | NA |
| 929 | 2001 | 11 | 163 | 84 | 2474 | 3 | 3 | 1 | 0 | NA |
| 930 | 2001 | 11 | 163 | 88 | 2020 | 3 | 2 | 2 | 1 | NA |
| 931 | 2001 | 11 | 163 | 88 | 2506 | 4 | 3 | 3 | 1 | NA |
| 932 | 2001 | 11 | 164 | 70 | 2047 | 5 | 0 | 1 | 0 | NA |
| 933 | 2001 | 11 | 166 | 78 | 942 | 3 | 3 | 1 | 0 | NA |
| 934 | 2001 | 11 | 166 | 98 | 2472 | 3 | 2 | 1 | 1 | NA |
| 935 | 2001 | 11 | 168 | 84 | 2454 | 3 | 0 | 3 | 1 | NA |
| 936 | 2001 | 11 | 170 | 124 | 2941 | 3 | 0 | 3 | 1 | NA |
| 937 | 2001 | 11 | 171 | 79 | 2613 | 3 | 0 | 3 | 1 | NA |
| 938 | 2001 | 11 | 172 | 90 | 2294 | 3 | 3 | 1 | 0 | NA |
| 939 | 2001 | 11 | 173 | 98 | 3754 | 3 | 2 | 3 | 1 | NA |
| 940 | 2001 | 11 | 174 | 95 | 2605 | 3 | 0 | 3 | 1 | NA |
| 941 | 2001 | 11 | 174 | 106 | 2972 | 3 | 3 | 1 | 0 | NA |
| 942 | 2001 | 11 | 175 | 97 | 3625 | 3 | 0 | 3 | 1 | NA |
| 943 | 2001 | 11 | 175 | 98 | 2466 | 3 | 2 | 1 | 0 | NA |
| 944 | 2001 | 11 | 176 | 95 | 3018 | 4 | 3 | 2 | 1 | NA |
| 945 | 2001 | 11 | 176 | 102 | 2755 | 3 | 2 | 1 | 0 | NA |
| 946 | 2001 | 11 | 177 | 92 | 2898 | 4 | 0 | 3 | 1 | NA |
| 947 | 2001 | 11 | 177 | 102 | 2822 | 4 | 3 | 2 | 1 | NA |
| 948 | 2001 | 11 | 177 | 116 | 3042 | 3 | 0 | 3 | 1 | NA |
| 949 | 2001 | 11 | 178 | 93 | 2882 | 4 | 3 | 3 | 1 | NA |
| 950 | 2001 | 11 | 178 | 119 | 3120 | 4 | 0 | 3 | 1 | NA |
| 951 | 2001 | 11 | 182 | 113 | 3417 | 3 | 0 | 3 | 1 | NA |
| 952 | 2001 | 11 | 183 | 116 | 3204 | 3 | 0 | 3 | 1 | NA |
| 953 | 2001 | 11 | 184 | 115 | 4286 | 3 | 2 | 2 | 1 | NA |
| 954 | 2001 | 11 | 184 | 117 | 3143 | 4 | 3 | 1 | 0 | NA |
| 955 | 2001 | 11 | 189 | 135 | 5245 | 4 | 0 | 3 | 1 | NA |
| 956 | 2001 | 11 | 189 | 137 | 5406 | 3 | 0 | 3 | 1 | NA |
| 957 | 2001 | 11 | 200 | 128 | 4628 | 3 | 3 | 3 | 1 | NA |
| 958 | 2001 | 12 | 154 | 72 | 1898 | 3 | 3 | 1 | 0 | NA |
| 959 | 2001 | 12 | 158 | 72 | 1982 | 3 | 3 | 1 | 0 | NA |
| 960 | 2001 | 12 | 159 | 78 | 2465 | 4 | 3 | 1 | 0 | NA |
| 961 | 2001 | 12 | 160 | 81 | 2772 | 3 | 3 | 1 | 0 | NA |
| 962 | 2001 | 12 | 166 | 84 | 2102 | 3 | 0 | 3 | 1 | NA |
| 963 | 2001 | 12 | 166 | 85 | 2686 | 3 | 0 | 3 | 1 | NA |
| 964 | 2001 | 12 | 166 | 86 | 2235 | 4 | 0 | 3 | 1 | NA |
| 965 | 2001 | 12 | 168 | 86 | 2914 | 3 | 3 | 3 | 1 | NA |
| 966 | 2001 | 12 | 168 | 86 | 3596 | 5 | 0 | 1 | 0 | NA |
| 967 | 2001 | 12 | 168 | 89 | 3458 | 3 | 2 | 1 | 1 | NA |
| 968 | 2001 | 12 | 169 | 89 | 2412 | 4 | 3 | 1 | 0 | NA |
| 969 | 2001 | 12 | 170 | 88 | 2306 | 4 | 3 | 1 | 0 | NA |
| 970 | 2001 | 12 | 174 | 92 | 7966 | 5 | 0 | 2 | 1 | 7434769 |
| 971 | 2001 | 12 | 174 | 94 | 2601 | 3 | 2 | 1 | 0 | NA |
| 972 | 2001 | 12 | 174 | 94 | 4491 | 4 | 3 | 1 | 0 | NA |
| 973 | 2001 | 12 | 174 | 96 | 2124 | 3 | 0 | 3 | 1 | NA |
| 974 | 2001 | 12 | 175 | 105 | 2858 | 3 | 0 | 3 | 1 | NA |
| 975 | 2001 | 12 | 176 | 93 | 2948 | 3 | 0 | 3 | 1 | NA |
| 976 | 2001 | 12 | 177 | 103 | 2401 | 3 | 3 | 2 | 1 | NA |
| 977 | 2001 | 12 | 178 | 99 | 2924 | 3 | 3 | 2 | 0 | NA |
| 978 | 2001 | 12 | 178 | 102 | 2862 | 3 | 0 | 3 | 1 | NA |
| 979 | 2001 | 12 | 179 | 98 | 2768 | 5 | 3 | 1 | 0 | 3264042 |
| 980 | 2001 | 12 | 179 | 102 | 3929 | 3 | 0 | 3 | 1 | NA |
| 981 | 2001 | 12 | 179 | 103 | 4124 | 3 | 3 | 1 | 0 | NA |
| 982 | 2001 | 12 | 180 | 103 | 3106 | 4 | 3 | 1 | 0 | NA |
| 983 | 2001 | 12 | 182 | 111 | 3281 | 4 | 3 | 1 | 0 | NA |
| 984 | 2001 | 12 | 183 | 115 | 4106 | 3 | 0 | 3 | 1 | NA |
| 985 | 2002 | 1 | 153 | 70 | 2077 | 4 | 3 | 1 | 1 | NA |
| 986 | 2002 | 1 | 155 | 70 | 1891 | 3 | 3 | 1 | 0 | NA |
| 987 | 2002 | 1 | 158 | 73 | 1990 | 3 | 2 | 1 | 0 | NA |
| 988 | 2002 | 1 | 158 | 75 | 2755 | 3 | 0 | 3 | 1 | NA |
| 989 | 2002 | 1 | 158 | 75 | 1189 | 3 | 2 | 1 | 0 | NA |
| 990 | 2002 | 1 | 158 | 79 | 2388 | 3 | 3 | 1 | 0 | NA |
| 991 | 2002 | 1 | 159 | 78 | 2274 | 3 | 0 | 3 | 1 | NA |
| 992 | 2002 | 1 | 160 | 83 | 1605 | 3 | 2 | 1 | 0 | NA |
| 993 | 2002 | 1 | 161 | 83 | 2498 | 3 | 3 | 2 | 1 | NA |
| 994 | 2002 | 1 | 162 | 75 | 1791 | 3 | 3 | 2 | 0 | NA |
| 995 | 2002 | 1 | 162 | 79 | 2718 | 4 | 3 | 1 | 0 | NA |
| 996 | 2002 | 1 | 162 | 80 | 2214 | 3 | 2 | 1 | 0 | NA |
| 997 | 2002 | 1 | 162 | 80 | 1483 | 3 | 3 | 1 | 0 | NA |
| 998 | 2002 | 1 | 162 | 81 | 3100 | 3 | 0 | 2 | 0 | NA |
| 999 | 2002 | 1 | 162 | 81 | 1247 | 3 | 2 | 2 | 1 | NA |
| 1000 | 2002 | 1 | 162 | 83 | 3519 | 3 | 3 | 2 | 1 | NA |
| 1001 | 2002 | 1 | 164 | 78 | 2742 | 4 | 3 | 1 | 0 | NA |
| 1002 | 2002 | 1 | 164 | 79 | 2235 | 3 | 3 | 2 | 1 | NA |
| 1003 | 2002 | 1 | 164 | 81 | 2435 | 4 | 3 | 1 | 0 | NA |
| 1004 | 2002 | 1 | 164 | 83 | 3123 | 3 | 3 | 1 | 1 | NA |
| 1005 | 2002 | 1 | 164 | 83 | 2428 | 3 | 3 | 3 | 0 | NA |
| 1006 | 2002 | 1 | 165 | 84 | 2339 | 4 | 3 | 1 | 0 | NA |
| 1007 | 2002 | 1 | 165 | 87 | 2420 | 3 | 2 | 1 | 0 | NA |
| 1008 | 2002 | 1 | 166 | 84 | 3243 | 3 | 3 | 1 | 0 | NA |
| 1009 | 2002 | 1 | 166 | 85 | 3182 | 3 | 3 | 1 | 0 | NA |
| 1010 | 2002 | 1 | 166 | 85 | 2633 | 3 | 3 | 1 | 0 | NA |
| 1011 | 2002 | 1 | 167 | 76 | 3821 | 5 | 0 | 1 | 0 | NA |
| 1012 | 2002 | 1 | 167 | 86 | 3261 | 3 | 0 | 3 | 1 | NA |
| 1013 | 2002 | 1 | 168 | 86 | 2354 | 3 | 2 | 1 | 0 | NA |
| 1014 | 2002 | 1 | 168 | 86 | 3066 | 3 | 3 | 1 | 0 | NA |
| 1015 | 2002 | 1 | 168 | 87 | 2969 | 3 | 3 | 1 | 0 | NA |
| 1016 | 2002 | 1 | 168 | 88 | 3096 | 4 | 3 | 1 | 0 | NA |
| 1017 | 2002 | 1 | 168 | 89 | 3846 | 3 | 2 | 1 | 0 | NA |
| 1018 | 2002 | 1 | 169 | 81 | 2676 | 3 | 2 | 1 | 0 | NA |
| 1019 | 2002 | 1 | 169 | 87 | 2725 | 3 | 0 | 3 | 1 | NA |
| 1020 | 2002 | 1 | 169 | 88 | 2543 | 3 | 3 | 1 | 0 | NA |
| 1021 | 2002 | 1 | 170 | 87 | 2247 | 4 | 3 | 1 | 0 | NA |
| 1022 | 2002 | 1 | 171 | 79 | 2320 | 3 | 2 | 1 | 1 | NA |
| 1023 | 2002 | 1 | 172 | 90 | 2986 | 3 | 3 | 1 | 0 | NA |
| 1024 | 2002 | 1 | 172 | 91 | 2425 | 3 | 3 | 1 | 0 | NA |
| 1025 | 2002 | 1 | 172 | 92 | 3765 | 4 | 3 | 1 | 0 | NA |
| 1026 | 2002 | 1 | 172 | 94 | 2378 | 3 | 3 | 2 | 1 | NA |
| 1027 | 2002 | 1 | 172 | 95 | 2197 | 3 | 3 | 2 | 1 | NA |
| 1028 | 2002 | 1 | 173 | 86 | 2506 | 4 | 3 | 1 | 0 | NA |
| 1029 | 2002 | 1 | 173 | 88 | 2956 | 4 | 3 | 1 | 0 | NA |
| 1030 | 2002 | 1 | 174 | 90 | 2786 | 4 | 3 | 1 | 1 | NA |
| 1031 | 2002 | 1 | 174 | 93 | 3145 | 3 | 0 | 3 | 1 | NA |
| 1032 | 2002 | 1 | 174 | 94 | 3580 | 3 | 2 | 3 | 1 | NA |
| 1033 | 2002 | 1 | 174 | 95 | 2094 | 3 | 3 | 2 | 1 | NA |
| 1034 | 2002 | 1 | 174 | 95 | 3009 | 4 | 3 | 1 | 0 | NA |
| 1035 | 2002 | 1 | 174 | 111 | 2188 | 3 | 3 | 1 | 1 | NA |
| 1036 | 2002 | 1 | 175 | 93 | 2787 | 3 | 2 | 1 | 0 | NA |
| 1037 | 2002 | 1 | 176 | 94 | 3245 | 3 | 3 | 1 | 1 | NA |
| 1038 | 2002 | 1 | 176 | 95 | 4026 | 4 | 0 | 3 | 1 | NA |
| 1039 | 2002 | 1 | 176 | 97 | 2846 | 4 | 3 | 1 | 0 | NA |
| 1040 | 2002 | 1 | 176 | 98 | 3688 | 3 | 3 | 1 | 0 | NA |
| 1041 | 2002 | 1 | 177 | 96 | 3833 | 3 | 0 | 3 | 1 | NA |
| 1042 | 2002 | 1 | 177 | 100 | 3642 | 4 | 3 | 1 | 1 | NA |
| 1043 | 2002 | 1 | 177 | 105 | 4104 | 3 | 2 | 1 | 0 | NA |
| 1044 | 2002 | 1 | 178 | 97 | 3281 | 3 | 2 | 2 | 1 | NA |
| 1045 | 2002 | 1 | 178 | 100 | 3674 | 3 | 2 | 1 | 0 | NA |
| 1046 | 2002 | 1 | 178 | 101 | 4021 | 4 | 3 | 1 | 0 | NA |
| 1047 | 2002 | 1 | 180 | 100 | 4022 | 3 | 3 | 1 | 0 | NA |
| 1048 | 2002 | 1 | 180 | 100 | 3232 | 3 | 3 | 1 | 0 | NA |
| 1049 | 2002 | 1 | 180 | 100 | 3025 | 5 | 3 | 1 | 0 | NA |
| 1050 | 2002 | 1 | 180 | 102 | 5146 | 3 | 3 | 1 | 1 | NA |
| 1051 | 2002 | 1 | 180 | 105 | 3362 | 3 | 2 | 1 | 0 | NA |
| 1052 | 2002 | 1 | 181 | 103 | 2963 | 4 | 3 | 1 | 0 | NA |
| 1053 | 2002 | 1 | 181 | 104 | 3966 | 4 | 3 | 2 | 0 | NA |
| 1054 | 2002 | 1 | 181 | 107 | 3524 | 4 | 3 | 1 | 0 | NA |
| 1055 | 2002 | 1 | 181 | 107 | 3621 | 5 | 3 | 1 | 0 | 3987543 |
| 1056 | 2002 | 1 | 182 | 107 | 3704 | 3 | 3 | 3 | 1 | NA |
| 1057 | 2002 | 1 | 183 | 110 | 3786 | 4 | 3 | 1 | 0 | NA |
| 1058 | 2002 | 1 | 184 | 112 | 2585 | 3 | 0 | 3 | 1 | NA |
| 1059 | 2002 | 1 | 184 | 113 | 4159 | 3 | 0 | 3 | 1 | NA |
| 1060 | 2002 | 1 | 184 | 114 | 4106 | 3 | 3 | 3 | 1 | NA |
| 1061 | 2002 | 1 | 185 | 114 | 5022 | 3 | 2 | 1 | 0 | NA |
| 1062 | 2002 | 1 | 185 | 115 | 5459 | 3 | 3 | 1 | 0 | NA |
| 1063 | 2002 | 1 | 187 | 116 | 3320 | 4 | 2 | 2 | 1 | NA |
| 1064 | 2002 | 1 | 187 | 120 | 4458 | 3 | 0 | 3 | 1 | NA |
| 1065 | 2002 | 1 | 189 | 125 | 4903 | 3 | 2 | 1 | 0 | NA |
| 1066 | 2002 | 1 | 190 | 115 | 3600 | 3 | 0 | 2 | 0 | NA |
| 1067 | 2002 | 1 | 193 | 136 | 4627 | 3 | 3 | 1 | 1 | NA |
| 1068 | 2002 | 1 | 194 | 119 | 2472 | 4 | 3 | 1 | 1 | NA |
| 1069 | 2002 | 1 | 194 | 144 | 2672 | 3 | 0 | 3 | 1 | NA |
| 1070 | 2002 | 1 | 197 | 140 | 5354 | 5 | 3 | 1 | 0 | 7281379 |
| 1071 | 2002 | 2 | 153 | 70 | 1289 | 3 | 3 | 1 | 0 | NA |
| 1072 | 2002 | 2 | 158 | 69 | 1523 | 3 | 3 | 1 | 0 | NA |
| 1073 | 2002 | 2 | 162 | 74 | 2042 | 3 | 2 | 2 | 0 | NA |
| 1074 | 2002 | 2 | 162 | 76 | 2419 | 3 | 2 | 1 | 1 | NA |
| 1075 | 2002 | 2 | 167 | 89 | 2894 | 3 | 3 | 1 | 0 | NA |
| 1076 | 2002 | 2 | 168 | 86 | 2844 | 4 | 3 | 1 | 0 | NA |
| 1077 | 2002 | 2 | 168 | 89 | 3028 | 4 | 2 | 1 | 0 | NA |
| 1078 | 2002 | 2 | 170 | 85 | 2827 | 4 | 0 | 2 | 1 | NA |
| 1079 | 2002 | 2 | 170 | 86 | 2183 | 3 | 3 | 1 | 0 | NA |
| 1080 | 2002 | 2 | 170 | 92 | 2024 | 3 | 3 | 2 | 0 | NA |
| 1081 | 2002 | 2 | 174 | 93 | 4261 | 3 | 3 | 1 | 0 | NA |
| 1082 | 2002 | 2 | 177 | 101 | 2146 | 3 | 3 | 1 | 1 | NA |
| 1083 | 2002 | 2 | 178 | 101 | 2630 | 3 | 0 | 3 | 1 | NA |
| 1084 | 2002 | 2 | 179 | 103 | 5471 | 3 | 3 | 1 | 0 | NA |
| 1085 | 2002 | 2 | 180 | 100 | 3342 | 3 | 3 | 2 | 1 | NA |
| 1086 | 2002 | 2 | 185 | 101 | 2396 | 3 | 2 | 1 | 0 | NA |
| 1087 | 2002 | 2 | 201 | 144 | 5193 | 3 | 3 | 1 | 1 | NA |
| 1088 | 2002 | 3 | 168 | 83 | 1877 | 4 | 3 | 1 | 0 | NA |
| 1089 | 2002 | 3 | 170 | 96 | 5535 | 5 | 3 | 1 | 0 | 12152617 |
| 1090 | 2002 | 3 | 172 | 84 | 2012 | 3 | 3 | 3 | 0 | NA |
| 1091 | 2002 | 3 | 173 | 90 | 2732 | 3 | 3 | 1 | 0 | NA |
| 1092 | 2002 | 3 | 177 | 103 | 3621 | 4 | 3 | 2 | 1 | NA |
| 1093 | 2002 | 3 | 179 | 116 | 3854 | 3 | 3 | 1 | 0 | NA |
| 1094 | 2002 | 3 | 182 | 109 | 2927 | 4 | 3 | 1 | 0 | NA |
| 1095 | 2002 | 3 | 185 | 105 | 3924 | 4 | 0 | 2 | 0 | NA |
| 1096 | 2002 | 3 | 186 | 107 | 4025 | 3 | 3 | 2 | 1 | NA |
| 1097 | 2002 | 3 | 186 | 110 | 3975 | 3 | 0 | 3 | 1 | NA |
| 1098 | 2002 | 3 | 191 | 114 | 3924 | 4 | 3 | 1 | 0 | NA |
| 1099 | 2002 | 3 | 191 | 121 | 5540 | 5 | 3 | 1 | 0 | 7684582 |
| 1100 | NA | NA | 170 | 109 | 1683 | 4 | 3 | 1 | 1 | NA |
| 1101 | NA | NA | 175 | 88 | 1891 | 3 | 0 | 4 | 1 | NA |
| 1102 | NA | NA | NA | NA | 4287 | 3 | 0 | 3 | 1 | NA |
| 1103 | NA | NA | NA | NA | 3499 | 3 | 0 | 3 | 1 | NA |
| 1104 | NA | NA | NA | NA | 2123 | 3 | 0 | 3 | 1 | NA |
| 1105 | NA | NA | NA | NA | 1707 | 3 | 2 | 2 | 1 | NA |
| 1106 | NA | NA | NA | NA | 2640 | 3 | 3 | 2 | 0 | NA |
| 1107 | NA | NA | NA | NA | 4525 | 3 | 3 | 3 | 1 | NA |
| 1108 | NA | NA | NA | NA | 2772 | 3 | NA | 2 | 0 | NA |
| 1109 | NA | NA | NA | NA | 2551 | 3 | NA | 3 | 1 | NA |
| 1110 | NA | NA | NA | NA | 3771 | 4 | 3 | 1 | 1 | NA |
| 1111 | NA | NA | NA | NA | 2867 | 4 | 3 | 2 | 0 | NA |
| 1112 | NA | NA | NA | NA | 3435 | 5 | 1 | 1 | 0 | NA |
| 1113 | NA | NA | NA | NA | 4143 | 5 | 1 | 2 | 1 | NA |
| 1114 | NA | NA | NA | NA | 4861 | 5 | 3 | 1 | 0 | NA |
| 1115 | NA | NA | NA | NA | 3118 | 5 | 3 | 3 | 1 | NA |

FL = fork length, BW = body weight, GW = ovary weight, MAGO = most advanced group of oocytes (1-5 = unyolked; early yolked; advanced yolked; migratory nucleus; hydrated), POF = postovulatory follicle (0-3 = absent; new; <12 hours old; 13-24 hours old). α stage atresia 1-5 = no atresia present, but advanced yolked oocytes are; <10% of advanced yolked oocytes are in the stage of atresia; 10-50% of advanced yolked oocytes are in the stage of atresia; >50% of advanced yolked oocytes are in the stage of atresia; 100% of advanced yolked oocytes are in the stage of atresia. β stage atresia 0-1 = absent; present. NA = not available.
